# Heparin therapy reduces 28-day mortality in adult severe sepsis patients: a systematic review and meta-analysis

**DOI:** 10.1186/s13054-014-0563-4

**Published:** 2014-10-16

**Authors:** Changsong Wang, Chunjie Chi, Lei Guo, Xiaoyang Wang, Libo Guo, Jiaxiao Sun, Bo Sun, Shanshan Liu, Xuenan Chang, Enyou Li

**Affiliations:** Department of Anesthesiology, First Affiliated Hospital of Harbin Medical University, No 23 Youzheng Street, Nangang District, Harbin, Heilongjiang 150001 China; Department of Anesthesiology, First Affiliated Hospital of Quan Zhou, No. 151 Yanjiang West Road, Yuexiu, Quan Zhou, Guangdong China; Department of Anesthesiology, Tianjin Huanhu Hospital, No. 122 Qixiangtai Road, Hexi, Tian Jin 300060 China

## Abstract

**Introduction:**

There are approximately 19 million new cases of sepsis worldwide each year. Among them, more than one quarter of patients die. We aimed to assess the effects of heparin on short-term mortality in adult patients with sepsis and severe sepsis.

**Methods:**

We searched electronic databases (Medline, Embase, and Cochrane Library databases; the Cochrane Controlled Trials Register) and conference proceedings (Web of Knowledge (Conference Proceedings Citation Index - Science, Conference Proceedings Citation Index - Social Sciences & Humanities)) from inception to July 2014, expert contacts and relevant websites. Controlled trials of heparin versus placebo in sepsis or severe sepsis were identified. In total two reviewers independently assessed eligibility, and four authors independently extracted data; consensus was reached by conference. We used the chi-square test and I^2^ to assess statistical heterogeneity (*P* <0.05). The primary analysis was based on the fixed-effect model to produce pooled odds ratios with 95% confidence intervals.

**Results:**

A total of nine publications were included in the meta-analysis. Heparin decreased 28-day mortality (n = 3,482, OR = 0.656, 95% CI = 0.562 to 0.765, *P* <0.0001). According to the meta-analysis of 28-day mortality, heterogeneity was not found among the eight randomized clinical trials (RCTs) (I^2^ = 0.0%). Heparin had no effect on bleeding events in sepsis (seven RCTs, n = 2,726; OR = 1.063; 95% CI = 0.834 to 1.355; *P* = 0.623; and I^2^ = 20.9%). Subgroup analysis demonstrated that the sample size may be a source of heterogeneity, but experimental design was not.

**Conclusions:**

Heparin may reduce 28-day mortality in patients with severe sepsis, at the same time, there was no increase in the risk of bleeding in the heparin group. We recommend the use of heparin for sepsis and severe sepsis.

**Electronic supplementary material:**

The online version of this article (doi:10.1186/s13054-014-0563-4) contains supplementary material, which is available to authorized users.

## Introduction

There are approximately 19 million new cases of sepsis worldwide each year. Among them, more than one quarter of patients die. In addition, there is an upward trend in sepsis incidence; sepsis and septic shock have become serious health problems [1,2]. Heparin was first applied in the treatment of sepsis in 1966. By evaluating a novel therapeutic concept in clinical practice, Martinez *et al*. found that lower mortality among sepsis patients may be related to the use of heparin, steroids, and vasoactive drugs [3], which inspired many researchers to evaluate heparin for sepsis treatment. Therefore, many clinical studies have been conducted since that first report. Currently, the efficacy and safety of heparin in sepsis patients remain controversial, with some authors suggesting that heparin may reduce 28-day mortality [4,5] and others reporting no effect on 28-day mortality [6-9]. We conducted a meta-analysis of studies on heparin treatment for sepsis to evaluate the effect of heparin on 28-day mortality and the occurrence of bleeding events in patients with sepsis.

## Methods

### Search strategy for identification of studies

**Figure 1:**
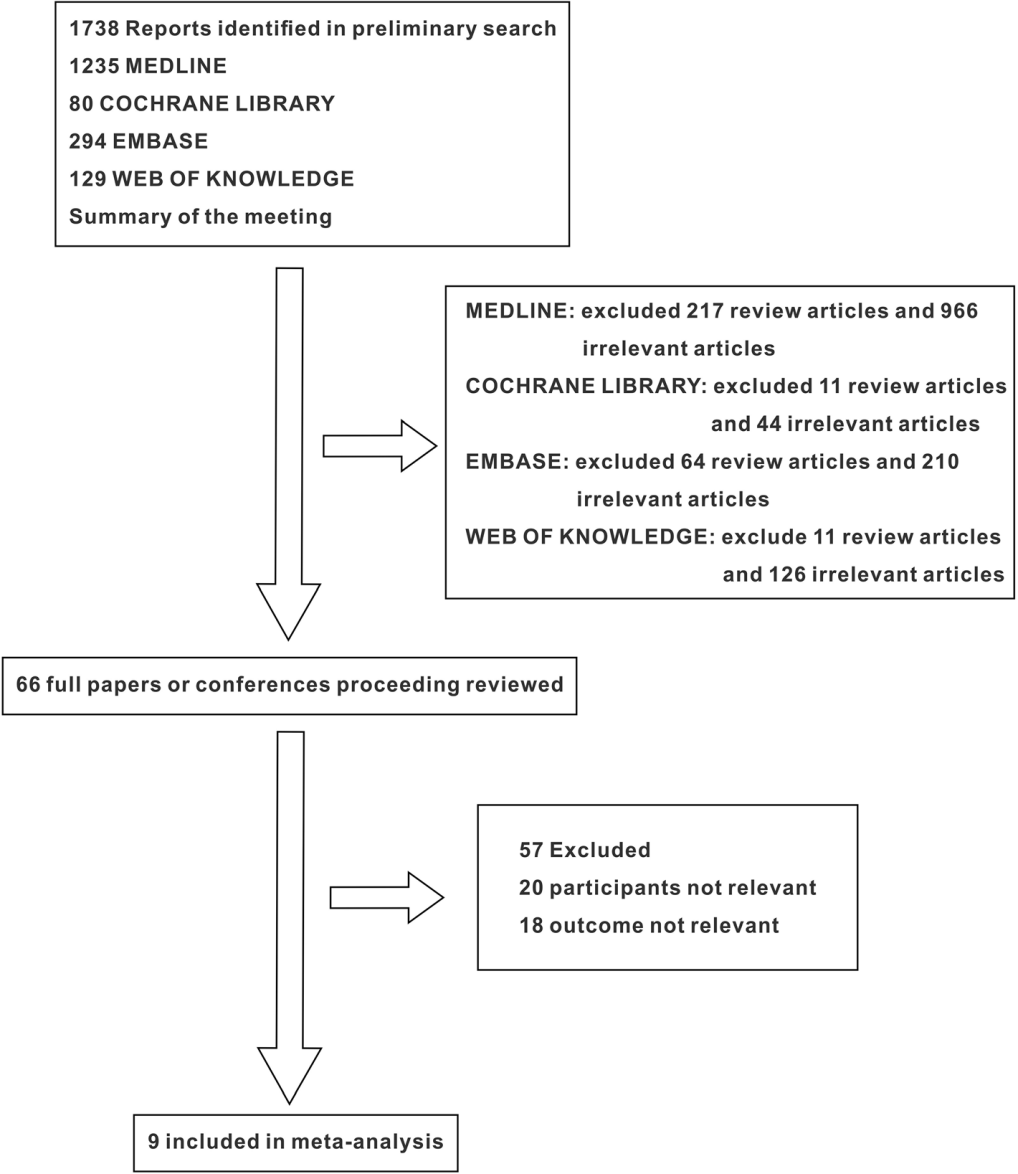
**Flow diagram of the literature search.**

We conducted a systematic review and several meta-analyses of the existing literature according to the methods recommended in the PRISMA statement for reporting systematic reviews and meta-analyses of studies that evaluate healthcare interventions (Figure 1). No institutional review board (IRB) approval or consents were needed for this systematic review because it evaluated published studies.

Our investigators were divided into four groups. CW was primarily responsible for the literature search group (CW and LG). JS and XW were responsible for the two literature review groups (JS, XW, LG, and XC). The trials were identified by electronic and manual searches. The electronic searches were performed by two authors (CW and LG) who independently searched the Medline, Embase, and Cochrane Library databases, the Cochrane Central Register of Controlled Trials, and Web of Knowledge (Conference Proceedings Citation Index - Science, Conference Proceedings Citation Index - Social Sciences & Humanities). We did not restrict our search based on language or year of publication. The last search update was July 2014. Additionally, we manually searched the Index Medicus of randomized clinical trials (RCTs), meta-analyses, and systematic reviews for studies that were missed in the initial electronic search. The search strategy identified 1,738 studies. Two literature review groups conducted the literature exclusion; 66 studies were included for potential interest.

### Inclusion and exclusion criteria

The literature inclusion and exclusion procedures were performed independently by two literature review groups (XW and XC; LG and EL). We excluded review, retrospective analyses, repeated literature reports, and repeated experiments (the same experiment analyzed and evaluated in different literature reports); purely physiological studies (for example, Effect of recombinant activated protein C and low-dose heparin on neutrophil-endothelial cell interactions in septic shock [10]); imaging studies; pediatric studies; studies on medications other than heparin; and studies without a control group (see Table S1 in Additional file 1). If data were missing, the literature search group contacted the authors for the relevant data. Subsequently, the two study review groups performed the initial verification. A disagreement occurred for two studies [5,9], which were eventually excluded after a discussion among all of the authors.

### Study selection and data abstraction

The data extraction strategy was discussed and designed by two authors (CW and CC). After all of the authors had discussed and reviewed the strategy, the corresponding author approved the final version of the study design strategy. The process yielded nine published studies. Two groups of researchers independently conducted a second round of data extraction from the literature. Key data were 28-day mortality and bleeding events. If the relevant data were missing or ambiguous, we contacted the authors for clarification. After the two separate literature review groups conducted the data extraction, the data were verified. If there was an inconsistency, the data extraction was repeated until a consensus was reached.

### Quality assessment

Quality assessments were performed separately by the two literature review groups. Studies that received inconsistent scores were scored again by all of the authors. The quality of the RCT studies was assessed using a modified Jadad scale [11], in which the generation of random sequences, blinding method, and reasons for withdrawal and dropout at the time of follow-up were evaluated. A seven-point scale was used, with one to three indicating a low-quality study and four to seven indicating a high-quality study. For non-randomized controlled trial (NRCT) studies, the data were extracted from subgroup analysis, and we used Silber’s quality assessment, which is a new score for subgroup analyses from randomized controlled trials [12] that includes prospectively collected data, subgroup analysis from a randomized controlled trial, multicenter trials (at least three centers), data from clinical events committees/data safety monitoring boards independent of steering committee monitoring (≥10%) and follow-up percentage. The maximum possible Silber score is five.

### Analyses

CC was responsible for the data analysis group (CC and JZ). The outcomes of the meta-analysis included 28-day mortality and bleeding events in patients with sepsis who were treated with heparin. After the extracted data were reviewed and verified by different study groups, Stata software (Version 12.0; StataCorp LP, College Station, TX, USA) was used for statistical analysis. The Cochrane Handbook of Systematic Reviews notes that the most commonly encountered effect measures used in clinical trials with dichotomous data are odds ratio (OR) and relative risk (RR). Both are entirely valid ways of describing a treatment. At the same time, because the mortality was calculated over different time points in most study designs, the hazard ratio (HR) is a more appropriate parameter for mortality calculations [13]. However, the HR may suffer relatively greater bias due to internal variability in different studies. As the value of the HR is considered closer to the OR, the authors chose ORs and 95% confidence intervals (CIs) as approximate parameters to evaluate the effect of heparin on 28-day mortality and bleeding events in septic patients [14,15].

Between-study heterogeneity was assessed using an I^2^ statistic (25% is defined as low heterogeneity, 50% as moderate heterogeneity, and 75% as high heterogeneity; significant heterogeneity is considered if I^2^ is greater than 50%) [16]. The fixed-effect model was applied if no or low significant heterogeneity was present, and pooled ORs were estimated using the Mantel-Haenszel method [17]. We performed a Z significance test on pooled ORs. Subgroup analysis was performed on bleeding events according to the sample size (that is, <100 or >100) and randomization method (that is, NRCT or RCT) of each study. We also assessed publication bias and small study bias. The Egger test was usually applied for continuous data analysis. However, the response variables of this study were dichotomous variables. Therefore, the publication bias was quantitatively examined using the Harbord test [18]. All statistical analyses are two-tailed tests. All hypotheses were tested at the alpha = 0.05 level.

## Results

Whether lower quality articles should be included in meta-analysis is still controversial [19]. Some think that the low scores do not mean low quality, and the study may be standard and have no bias [20]. In other words, the research process may strictly regulate, but the paper is not standardized [21]. In an empirical study of the relation of quality scores to treatment differences in published meta-analysis of seven groups of controlled randomized clinical trials comprising 107 primary studies, Emerson *et al*. found no relation between treatment difference and overall quality score [22]. For the above reasons and PRISMA principles, we analyzed the data including and ruling out low-quality articles, and we found that after removing the low-quality articles, they had no effect on the results. Because the more studies integrated into the article, the greater the sample size, the higher the performance test, the body of the article is analysis of all of the studies. In addition, the analysis of the high-quality studies is in Table S2 in Additional file 1.

Nine studies (Table 1) were included in the meta-analysis [4,7,8,23-28]. Except for a lack of 28-day mortality data in Yang *et al.*’s study [27], the remaining eight studies all performed analyses of 28-day mortality. Additionally, Liu *et al.*’s study [28] had no bleeding events data. Raw data were directly provided in eight [4,7,8,23-25,27,28] of the nine studies. The data from one study [26] were obtained indirectly from the corresponding authors [29]. The abstract of Abraham *et al.*’s study [25] indicated that 28-day mortality was the primary outcome; however, the specific time point was not shown in the results and tables. We included this study for 28-day mortality analysis after discussion. Seven studies [4,7,8,23-25,27] were included in the meta-analysis of bleeding events.

**Table 1:** **Characteristics of included trials**

**Article**	**Number of patients**	**Number of research centers**	**Sepsis severity**	**APACHE II scores**	**Design**	**Intervention**	**Quality assessment**	**Outcome**	
Warren *et al*. [24]	1157	211	Severe sepsis	49 (16)^*^	NRCT	Study group: Unfractionated or low-molecular-weight heparin for venous thrombosis prophylaxis (≤10000 IU subcutaneous per day), and heparin flushes for vascular catheter patency (IV of ≤ 2 IU per kilogram of body weight per hour). Control group: 1% human albumin.	5 (Silber)	Mortality (28-d) Bleeding effects	Prophylactic
Bernard *et al. *[26]	840	164	Severe sepsis	25 (7.8)	NRCT	Study group: a dose of unfractionated heparin of up to 15,000 U per day. Control group: 0.9% saline with or without 0.1% human serum albumin.	5 (Silber)	Mortality (28-d)	Prophylactic
Abraham *et al.* [25]	992	245	Severe sepsis	25 (7.2)	NRCT	Study group: at least 1 dose of unfractionated heparin or low-molecular-weight heparin for 120 hours. Control group: arginine citrate buffer.	5 (Silber)	Mortality (28-d)	Prophylactic
								Bleeding effects	
Ai *et al*. [8]	40	1	Sepsis	15 (4.1)	RCT	Study group: low-molecular-weight heparin 5000 IU subcutaneous per 12 hours for 7 days. Control group: placebo.	1 (Jadad)	Mortality (28-d )	Prophylactic
								Bleeding effects	
Zhang *et al.* [7]	22	1	Severe sepsis	__	RCT	Study group: IV heparin (3 ~ 4) U/kg/h for 7 days. Control group: placebo.	3 (Jadad)	Mortality (28-d)	Prophylactic
								Bleeding effects	
Jaimes *et al*. [23]	317	1	Sepsis	9.5 (1.7)	RCT	Study group: Unfractionated heparin 500 units/hour for 7 days. Control group: placebo.	7 (Jadad)	Mortality (28-d)	Prophylactic
								Bleeding effects	
Zhao *et al*. [4]	79	1	Sepsis	15 (5.3)	RCT	Study group: heparin sodium IVGTT of 40 ~ 50 mg/d for 5–7 days. Control group: placebo.	4 (Jadad)	Mortality (28-d)	Prophylactic
								Bleeding effects	
Yang *et al.* [27]	119	1	Sepsis	__	RCT	Study group: heparin sodium IVGTT of 2 mg/kg/d. Control group: 0.9% saline.	5 (Jadad)	Bleeding effects	Prophylactic
Liu *et al.* [28]	37	1	Sepsis	20 (6.2)	RCT	Study group: 70 U/kg/24 h heparin was administered by continuous infusion for 5–7 days	3 (Jadad)	Mortality (28-d)	Prophylactic
						Control group: saline.			

^*^Simplified acute physiology and chronic health evaluation score version II. IV, intravenous; IVGTT, intravenous glucose-tolerance test.

### Twenty-eight-day mortality analysis

We performed analyses for eight studies [4,7,8,23-26,28], which included 3,482 participants (2,378 participants were included in the patient group and 1,104 participants in the control group). Within 28 days of admission, 722 (30.36%) died in the patient group, and 420 (38.04%) died in the control group (OR = 0.656; 95% CI = 0.562 to 0.765; *P* <0.0001; I^2^ = 0.0%), indicating a statistically significant reduction in 28-day mortality in heparin-treated patients with sepsis (see Table S3 in Additional file 1 and Additional file 2). There was no evidence of between-study heterogeneity (I^2^ = 0.0%), and a sensitivity analysis was not performed.

### Subgroup analysis

Subgroup analysis was performed according to the different experimental designs of the eight studies. The included studies were divided into two subgroups: an NRCT group and an RCT group. For the three NRCT studies, OR = 0.648, 95% CI = 0.550 to 0.764, *P* <0.001, and I^2^ = 6.7%. For the five RCT studies, OR = 0.717, 95% CI = 0.458 to 1.122, *P* = 0.145, and I^2^ = 0.0%. Subgroup analysis indicated that the results of the NRCT group reached statistical significance. Although the results from the RCT group are not statistically significant, the high value of the 95% CIs (1.122) is very close to the invalid line. When heterogeneity was assessed in the different subgroups, there was either low-level or no heterogeneity (see Table S3 in Additional file 1 and Additional file 3).

**Figure 2:**
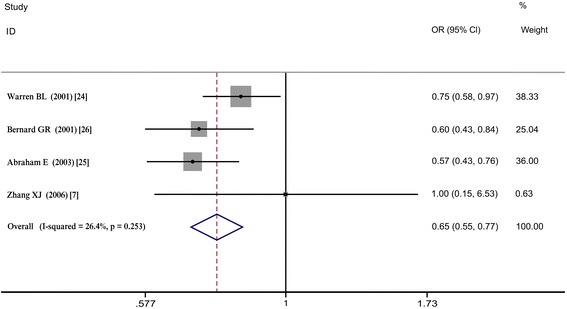
**Subgroup analysis of 28-day mortality according to sepsis severity (the severe sepsis group).**

Subgroup analysis was also performed according to sepsis severity. For the four studies on severe sepsis (defined as sepsis complicated by organ dysfunction and tissue hypoperfusion), OR = 0.650, 95% CI = 0.552 to 0.766, *P* <0.001, and I^2^ = 0%. For the four studies on non-severe sepsis, OR = 0.702, 95% CI = 0.4443 to 1.115, *P* = 0.134, and I^2^ = 0.0%. Heparin may have therapeutic effects in patients with severe sepsis, but it has no effect on patients with non-severe sepsis. Similar to the subgroup analysis of the different experimental designs, the high 95% CIs from the sepsis group were also very close to the invalid line (see Figure 2 and Table S3 in Additional file 1).

### Publication bias analysis

We also analyzed publication bias for 28-day mortality in the included studies. Because the response variables were dichotomous, publication bias was quantitatively examined using the Harbord test. The *P* value was 0.881 in the 28-day mortality analysis, indicating that there was no evidence of publication bias in these studies (see Table S3 in Additional file 1 and Additional file 4).

### The bleeding events analysis

**Figure 3:**
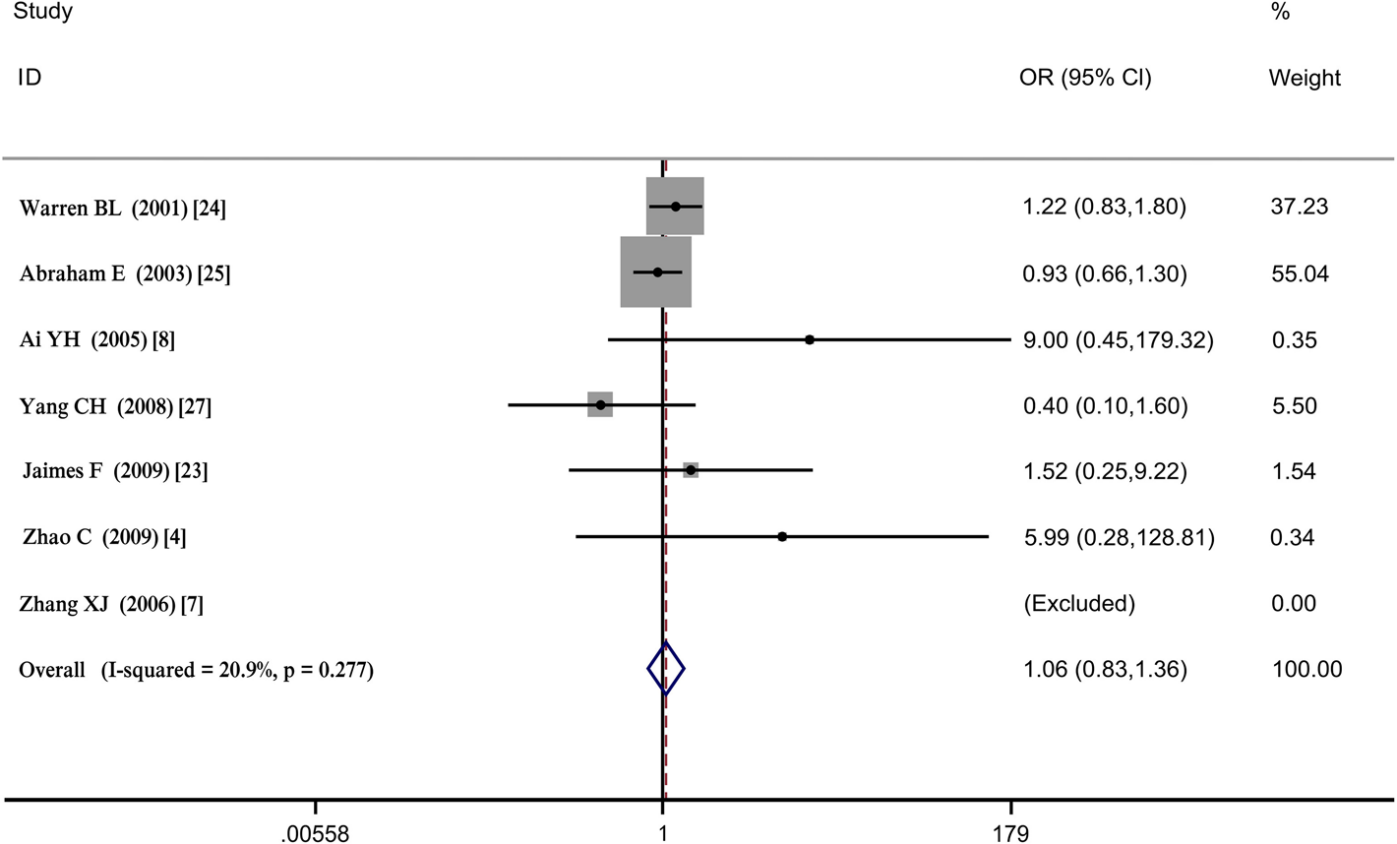
**Bleeding events.**

We performed a statistical analysis on seven studies [4,7,8,23-25,27] that included 2,726 participants (1,775 participants in the patient group and 951 participants in the control group). There were 251 (14.14%) bleeding events in the patient group and 112 (11.78%) in the control group (OR = 1.063; 95% CI = 0.834 to 1.355; *P* = 0.623; I^2^ = 20.9%). The results failed to reach statistical significance, indicating that heparin has no effect on bleeding events in patients with sepsis (see Figure 3 and Table S3 in Additional file 1).

There was a low level of heterogeneity between studies, and a sensitivity analysis was not performed.

### Subgroup analysis

To investigate whether the bleeding events were associated with sepsis severity, we conducted a subgroup analysis according to sepsis severity. For the severe sepsis group, OR = 1.076, 95% CI = 0.836 to 1.384, *P* = 0.569, I^2^ = 34.9%; for the sepsis group, OR = 0.897, 95% CI = 0.354 to 2.275, *P* = 0.819, I^2^ = 35.1%. The statistical results did not show that bleeding events were related to sepsis severity (see Table S3 in Additional file 1 and Additional file 5). A subgroup analysis was performed to investigate the effects of different subgroups on heterogeneity. The included studies were divided into two subgroups: an NRCT group and an RCT group. For the two NRCT studies, OR = 1.045, 95% CI = 0.802 to 1.362, *P* = 0.745, I^2^ = 7.4%. For the four RCT studies (Zhang *et al.* [7] was automatically excluded), OR = 1.524, 95% CI = 0.369 to 6.292, *P* = 0.561, and I^2^ = 43.4%. Four studies with sample sizes over 100 were included in this subgroup with an OR = 1.018, 95% CI = 0.796 to 1.302, *P* = 0.887, and I^2^ = 1.0%. Two studies (Zhang *et al.* [7] was automatically excluded) were included in the subgroup with sample sizes less than 100 with an OR = 7.497, 95% CI = 0.885 to 63.529, *P* = 0.065, I^2^ = 0.0%. (see Table S3 in Additional file 1, Additional file 6 and Additional file 7).

Heterogeneity decreased in the NRCT subgroup, while heterogeneity increased in the sample size and RCT subgroups, compared with overall heterogeneity. Therefore, sample size may be a source of heterogeneity.

### Publication bias analysis

The *P* value was 0.343 in the bleeding events analysis (see Table S3 in Additional file 1 and Additional file 8).

## Discussion

This study revealed that heparin can significantly reduce 28-day mortality in patients with severe sepsis but that it has no effect on the occurrence of bleeding events.

During the process of the analysis, two researchers independently extracted the data. If the study data were inconsistent, we re-collected the data after discussion. We also contacted the corresponding authors to obtain detailed information or to confirm some key data regarding experimental results. This study design can reduce ascertainment bias [30].

Of the nine articles, heparin was the objective drug in six studies. Although the effects of heparin were not the objectives of three original articles, heparin was not a concomitant medication in our extracted data. The three original studies were large, multicenter RCTs [24-26] of antithrombin III (randomized to the ‘antithrombin III group’ , ‘placebo group’), tissue factor pathway inhibitor (TFPI) (randomized to the ‘drotrecogin alfa (activated) group’ , ‘placebo group’), and recombinant human activated protein C (randomized to the ‘tifacogin group’ , ‘placebo group’) for the treatment of severe sepsis. Heparin was used in all of these large RCT studies; however, it was only used as a concomitant medication. The authors did not evaluate the efficacy of heparin on sepsis treatment separately. In the above three studies, the author lists the proportion of patients using heparin in the ‘experimental group’ and the ‘placebo group’. When we extracted the data from the above three studies, with reference to Agarwal *et al.*’s method [29], the patients who received heparin in the ‘placebo group’ were treated as the study group, and the patients in the ‘placebo group’ who did not receive heparin were treated as the control group. Only data from a blank group were extracted for analysis to eliminate the interference of other anticoagulants. Both Levi *et al.*’s study [5] and Iba *et al.*’s study [9] investigated heparin treatment for sepsis. In addition to heparin, the patients also received drotrecogin alfa (activated) (Drot AA) or antithrombin, respectively. In contrast with the Warren *et al.* [24], Abraham *et al*. [25], and Bernard *et al*. [26] trials, we were not able to extract data about the blank group without other anticoagulants. The studies indicated that Drot AA has antithrombotic and profibrinolytic properties, which can provide adequate prevention from venous thromboembolism itself. However, Drot AA can potentially weaken the preventive effect of heparin in sepsis treatment [5,26], which may affect the study results. Therefore, after discussion, we did not include these studies in the analysis.

Agarwal *et al*. [29] applied RevMan statistical software to perform a simple meta-analysis on the Warren *et al*. [24], Abraham *et al*. [25], and Bernard *et al*. [26] studies to investigate the efficacy of heparin therapy for patients with sepsis. The study result was relatively consistent with our study, suggesting that heparin can significantly reduce 28-day mortality in patients with severe sepsis. In contrast with Agarwal *et al*.’s study, we included four additional studies that described RCTs evaluating heparin treatment. More importantly, we included a new study conducted in 2009 [23], which was considered the first heparin RCT with a large sample size and a blank control. Therefore, the inclusion of this study undoubtedly improved the reliability of the meta-analysis as it played a prominent role in the study analysis. In addition, Agarwal *et al*.’s study did not perform a meta-analysis on the effect of heparin on the occurrence of bleeding events. However, we performed a meta-analysis of seven studies and found that heparin has no effect on bleeding events in patients with sepsis. The reason may be that the low dose of heparin in the original articles leads to no increase in the risk of bleeding in patients treated with heparin.

In this study, we found that heparin can reduce 28-day mortality in patients with severe sepsis; however, it had no effect on 28-day mortality in patients with non-severe sepsis. A meta-analysis of heparin therapy in medically critical surgical patients [31] revealed that heparin can effectively prevent deep vein thrombosis (DVT) without increasing the risk of bleeding. The latest published Surviving Sepsis Campaign (SSC) [2] guideline also recommends the use of heparin as part of DVT prophylaxis for severe sepsis. The results of this study showed that heparin reduced 28-day mortality in patients with severe sepsis. The possible explanations are as follows: 1. As an anticoagulant, heparin effectively reduces the occurrence of deep venous thromboembolism in patients with severe sepsis. 2. The occurrence and development of sepsis are closely related to inflammation and the coagulation system [6,32]. The interaction between coagulation activation and the inflammatory response is the characteristic pathological process of sepsis [33,34]. As an anticoagulant, heparin also has an anti-inflammatory effect: it can reduce the blood levels of inflammatory mediators (such as histamine) and can increase the release of TFPIs [35]. Heparin plays an anti-inflammatory role in the pathophysiology of severe sepsis [36].

This study also has limitations. The *New England Journal of Medicine* published a study of multicenter, randomized placebo-controlled studies in 2011 [37] that showed no differences in the prevention of DVT between unfractionated heparin and low-molecular-weight heparin. However, a meta-analysis by Alhazzani *et al*. [31] revealed that low-molecular-weight heparin can prevent thrombosis better than unfractionated heparin. Low-molecular-weight heparin can also effectively reduce the incidence of DVT. Moreover, studies have revealed different anticoagulant mechanisms for thrombin inhibition between unfractionated heparin and low-molecular-weight heparin [38].We attempted to perform a subgroup analysis on the two types of heparin. However, because the existing studies of heparin therapy, except for the XPRESS study [5], did not differentiate the two types, our study could not compare the effectiveness of the different types of heparin treatment for patients with sepsis. In NRCT studies [24-26], heparin was administered to some patients and not to others at the discretion of the treating physician. This confounding factor may have an effect on the results of the meta-analysis. After careful observation, we found that the 95% CIs of the RCT and sepsis groups were very close to the invalid lines in the subgroup analysis of 28-day mortality, meaning that there is a trend of decreased short-term mortality in patients who used heparin. Large-sample-size, multicenter, double-blind, parallel-group trials are needed to validate whether heparin can significantly reduce 28-day mortality.

## Conclusions

In conclusion, heparin may reduce 28-day mortality in patients with severe sepsis because there was a trend of decreased short-term mortality in patients who used heparin. At the same time, there was no increase in the risk of bleeding in the heparin group. We recommend the use of heparin for sepsis and severe sepsis.

## Key messages

- In adults, the efficacy of heparin for sepsis has not been well established.
- This meta-analysis evaluated the short-term effects of heparin on sepsis mortality. Pooled data showed that heparin may decrease 28-day mortality and longer-term mortality.
- The use of heparin for sepsis is safe with no increase in the risk of bleeding.

## Additional files

Additional file 1: Table S1. Excluded literature. **Table S2.** Meta-analysis of high-quality articles. **Table S3.** Outcome effect estimates. Additional file 2:
**Forest plot of 28-day mortality.**
Additional file 3:
**Subgroup analysis of 28-day mortality (according to the different experimental designs).**
Additional file 4:
**The Harbord plot for 28-day mortality.**
Additional file 5:
**Subgroup analysis hemorrhagic events (according to sepsis severity).**
Additional file 6:
**Subgroup analysis hemorrhagic events (according to the different experimental designs).**
Additional file 7:
**Subgroup analysis hemorrhagic events (according to sample size).**
Additional file 8:
**The Harbord plot for hemorrhagic events.**

## Abbreviations

CIs: confidence intervals
Drot AA: drotrecogin alfa (activated)
DVT: deep vein thrombosis
HR: hazard ratio
NRCT: nonrandomized controlled trial
OR: odds ratio
RCT: randomized controlled trial
RR: relative risk
SSC: Surviving Sepsis Campaign
TFPI: tissue factor pathway inhibitor
