# Supplementary material for: Heparin therapy reduces 28-day mortality in adult severe sepsis patients: a systematic review and meta-analysis
Source: Crit Care. 2014 Oct 16;18(5):563. doi: 10.1186/s13054-014-0563-4 (PMC4213495; doi:10.1186/s13054-014-0563-4)
Supplement: Additional file 1: Table S1. — Excluded literature. Table S2. Meta-analysis of high-quality articles. Table S3. Outcome effect estimates. [file 13054_2014_563_MOESM1_ESM.doc]

**Heparin Therapy Reduces 28-Day Mortality in Adult Severe Sepsis Patients—a systematic review and meta-analysis**

Changsong Wang, MD^1^, Chunjie Chi, MS^1^, Lei Guo, MD^1^, Xiaoyang Wang, MS^1^, Libo Guo, MS^1^, Jiaxiao Sun, MS^2^, Bo Sun, MS^1^, Shanshan Liu, MS^1^, Xuenan Chang, MS^3^, Enyou Li, MD^1^.

Additional file 1 Excluded literature

| Year | Authors | Exclued Reason |
| --- | --- | --- |
| 1966 | [Martinez JT](http://www.ncbi.nlm.nih.gov/pubmed?term=Martinez JT%5BAuthor%5D&cauthor=true&cauthor_uid=5325605) [1] | Study objective did not match, no heparin control group, no relevant outcome |
| 1968 | Holland P [2] | Study objective did not match |
| 1969 | Masera G [3] | Study objective did not match |
| 1969 | Masera G [4] | Study objective did not match |
| 1970 | JAMES J [5] | Study objective did not match |
| 1971 | Popov S [6] | No relevant outcome |
| 1972 | Wilson FE [7] | Study objective did not match |
| 1973 | Blum D [8] | Study objective did not match |
| 1973 | Hathaway WE [9] | Study objective did not match |
| 1973 | Mitterstieler G [10] | Study objective did not match |
| 1974 | de Aquino-Jaso ME [11] | Study objective did not match |
| 1977 | Cavanagh D [12] | Study objective did not match |
| 1978 | Maszkiewicz W [13] | Study objective did not match |
| 1978 | Schippan R [14] | Study objective did not match |
| 1978 | Iturriaga Ruiz S [15] | Study objective did not match |
| 1979 | Wytrychowski M [16] | Study objective did not match |
| 1981 | Abizanda Campos R [17] | No relevant data |
| 1982 | Chaĭtsev VG [18] | No relevant data |
| 1982 | Grygalewicz J [19] | Study objective did not match |
| 1983 | Taenaka N [20] | No relevant outcome |
| 1983 | Lytkin MI [21] | No relevant data |
| 1983 | Haneberg B [22] | Study objective did not match |
| 1983 | Godula-Stuglik U [23] | Study objective did not match |
| 1984 | Dobrovol'skiĭ VI [24] | No relevant data |
| 1997 | [Smith OP](http://www.ncbi.nlm.nih.gov/pubmed?term=Smith OP%5BAuthor%5D&cauthor=true&cauthor_uid=9393338) [25] | Control group deficient |
| 1997 | Ghanem J [26] | No relevant outcome |
| 1997 | M.Hofmann [27] | No relevant outcome |
| 1999 | [Boldt J](http://www.ncbi.nlm.nih.gov/pubmed?term=Boldt J%5BAuthor%5D&cauthor=true&cauthor_uid=9921711) [28] | No relevant outcome |
| 2002 | [Rublee D](http://www.ncbi.nlm.nih.gov/pubmed?term=Rublee D%5BAuthor%5D&cauthor=true&cauthor_uid=12225612) [29] | No relevant outcome |
| 2002 | Messori A [30] | No relevant outcome |
| 2002 | Davidson BL [31] | Communicate with the author |
| 2003 | [Derhaschnig U](http://www.ncbi.nlm.nih.gov/pubmed?term=Derhaschnig U%5BAuthor%5D&cauthor=true&cauthor_uid=12682480) [32] | Study objective did not match,no relevant outcome |
| 2005 | [Koestenberger M](http://www.ncbi.nlm.nih.gov/pubmed?term=Koestenberger M%5BAuthor%5D&cauthor=true&cauthor_uid=16188810) [33] | No relevant outcome |
| 2005 | [Agarwal R](http://www.ncbi.nlm.nih.gov/pubmed?term=Agarwal R%5BAuthor%5D&cauthor=true&cauthor_uid=16007415) [34] | The corresponding authors, No relevant data |
| 2005 | [Gullo A](http://www.ncbi.nlm.nih.gov/pubmed?term=Gullo A%5BAuthor%5D&cauthor=true&cauthor_uid=16288186) [35] | No relevant outcome, no heparin control group |
| 2006 | [Jaimes F](http://www.ncbi.nlm.nih.gov/pubmed?term=Jaimes F%5BAuthor%5D&cauthor=true&cauthor_uid=16729879) [36] | No relevant outcome |
| 2006 | [Jaimes F](http://www.ncbi.nlm.nih.gov/pubmed?term=Jaimes F%5BAuthor%5D&cauthor=true&cauthor_uid=16729879) [37] | Repeated literature |
| 2006 | 窗体顶端  [Hoffmann JN](http://www.ncbi.nlm.nih.gov/pubmed?term=Hoffmann JN%5BAuthor%5D&cauthor=true&cauthor_uid=16676077) [38]窗体底端 | Secondary analysis |
| 2006 | 窗体顶端  [Kirschenbaum LA](http://www.ncbi.nlm.nih.gov/pubmed?term=Kirschenbaum LA%5BAuthor%5D&cauthor=true&cauthor_uid=16775572) [39]窗体底端 | No relevant outcome |
| 2006 | 窗体顶端  [Wiedermann CJ](http://www.ncbi.nlm.nih.gov/pubmed?term=Wiedermann CJ%5BAuthor%5D&cauthor=true&cauthor_uid=16424704) [40]窗体底端 | Secondary analysis |
| 2006 | 窗体顶端  [Kienast J](http://www.ncbi.nlm.nih.gov/pubmed?term=Kienast J%5BAuthor%5D&cauthor=true&cauthor_uid=16409457) [41]窗体底端 | Secondary analysis |
| 2007 | Zarychanski, R [42] | No relevant data |
| 2007 | D. Dries [43] | The experiment report |
| 2007 | 窗体顶端  [Saito H](http://www.ncbi.nlm.nih.gov/pubmed?term=Saito H%5BAuthor%5D&cauthor=true&cauthor_uid=17059423) [44]窗体底端 | No relevant outcome |
| 2007 | 窗体顶端  [Pechlaner C](http://www.ncbi.nlm.nih.gov/pubmed?term=Pechlaner C%5BAuthor%5D&cauthor=true&cauthor_uid=17765516) [45]窗体底端 | Communicate with the author |
| 2007 | 窗体顶端  [Levi M](http://www.ncbi.nlm.nih.gov/pubmed?term=Levi M%5BAuthor%5D&cauthor=true&cauthor_uid=17556722) [46]窗体底端 | Background medication |
| 2008 | 窗体顶端  [Eid A](http://www.ncbi.nlm.nih.gov/pubmed?term=Eid A%5BAuthor%5D&cauthor=true&cauthor_uid=18931224) [47]窗体底端 | Secondary analysis |
| 2008 | 窗体顶端  [Zarychanski R](http://www.ncbi.nlm.nih.gov/pubmed?term=Zarychanski R%5BAuthor%5D&cauthor=true&cauthor_uid=18824906) [48]窗体底端 | Secondary analysis |
| 2008 | 窗体顶端  [Agarwal R](http://www.ncbi.nlm.nih.gov/pubmed?term=Agarwal R%5BAuthor%5D&cauthor=true&cauthor_uid=18250355) [49]窗体底端 | Communicate with the author,secondary analysis |
| 2009 | 窗体顶端  [Levy M](http://www.ncbi.nlm.nih.gov/pubmed?term=Levy M%5BAuthor%5D&cauthor=true&cauthor_uid=19367397) [50]窗体底端 | Secondary analysis |
| 2009 | 窗体顶端  [Angstwurm M](http://www.ncbi.nlm.nih.gov/pubmed?term=Angstwurm M%5BAuthor%5D&cauthor=true&cauthor_uid=19189066) [51]窗体底端 | Secondary analysis |
| 2009 | 窗体顶端  [Tsen A](http://www.ncbi.nlm.nih.gov/pubmed?term=Tsen A%5BAuthor%5D&cauthor=true&cauthor_uid=18636042) [52]窗体底端 | No relevant outcome |
| 2009 | 窗体顶端  [Shorr AF](http://www.ncbi.nlm.nih.gov/pubmed?term=Shorr AF%5BAuthor%5D&cauthor=true&cauthor_uid=19132200) [53]窗体底端 | Secondary analysis |
| 2012 | 窗体顶端  [Iba T](http://www.ncbi.nlm.nih.gov/pubmed?term=Iba T%5BAuthor%5D&cauthor=true&cauthor_uid=22542365) [54]窗体底端 | Background medication |
| 2012 | 窗体顶端  [Han XD](http://www.ncbi.nlm.nih.gov/pubmed?term=Han XD%5BAuthor%5D&cauthor=true&cauthor_uid=22248749) [55]窗体底端 | No relevant outcome |
| 2012 | Gamzatov KhA [56] | No relevant data |

1. [Martinez JT](http://www.ncbi.nlm.nih.gov/pubmed?term=Martinez JT%5BAuthor%5D&cauthor=true&cauthor_uid=5325605), [Fernandez G](http://www.ncbi.nlm.nih.gov/pubmed?term=Fernandez G%5BAuthor%5D&cauthor=true&cauthor_uid=5325605), [Vazquez-Leon H](http://www.ncbi.nlm.nih.gov/pubmed?term=Vazquez-Leon H%5BAuthor%5D&cauthor=true&cauthor_uid=5325605). Clinical evaluation of new therapeutic concepts in septic shock. [Obstet Gynecol](http://www.ncbi.nlm.nih.gov/pubmed/5325605#%23) 1966;27(2):296–301.
2. [Holland P](http://www.ncbi.nlm.nih.gov/pubmed?term=Holland P%5BAuthor%5D&cauthor=true&cauthor_uid=5681136), [Bobo MF](http://www.ncbi.nlm.nih.gov/pubmed?term=Bobo MF%5BAuthor%5D&cauthor=true&cauthor_uid=5681136), [Shackelford E](http://www.ncbi.nlm.nih.gov/pubmed?term=Shackelford E%5BAuthor%5D&cauthor=true&cauthor_uid=5681136). Meningococcemia and intravascular clotting: the use of heparin in management. [J Ky Med Assoc](http://www.ncbi.nlm.nih.gov/pubmed/?term=Meningococcemia+and+intravascular+clotting%3A+the+use+of+heparin+in+management.J+Ky+Med+Assoc.+1968#%23) 1968;66(9):807-810
3. [Masera G](http://www.ncbi.nlm.nih.gov/pubmed?term=Masera G%5BAuthor%5D&cauthor=true&cauthor_uid=5795994), [Carnelli V](http://www.ncbi.nlm.nih.gov/pubmed?term=Carnelli V%5BAuthor%5D&cauthor=true&cauthor_uid=5795994). Heparin therapy in meningococcal sepsis. [Minerva Pediatr](http://www.ncbi.nlm.nih.gov/pubmed/?term=Heparin+therapy+in+meningococcal+sepsis1969Minerva+Pediatr.+1969+Feb+25%3B21(8)%3A321.#%23) 1969;21(8):321.
4. [Masera G](http://www.ncbi.nlm.nih.gov/pubmed?term=Masera G%5BAuthor%5D&cauthor=true&cauthor_uid=5777642), [Carnelli V](http://www.ncbi.nlm.nih.gov/pubmed?term=Carnelli V%5BAuthor%5D&cauthor=true&cauthor_uid=5777642). Therapy with heparin in meningococcic sepsis [Minerva Pediatr](http://www.ncbi.nlm.nih.gov/pubmed/?term=Therapy+with+heparin+in+meningococcic+sepsisMinerva+Pediatr.+1969+Mar+10%3B21(10)%3A394-401.#%23) 1969;21(10):394-401.
5. [Corrigan JJ Jr](http://www.ncbi.nlm.nih.gov/pubmed?term=Corrigan JJ Jr%5BAuthor%5D&cauthor=true&cauthor_uid=4989565), [Jordan CM](http://www.ncbi.nlm.nih.gov/pubmed?term=Jordan CM%5BAuthor%5D&cauthor=true&cauthor_uid=4989565). Heparin therapy in septicemia with disseminated intravascular coagulation. [N Engl J Med](http://www.ncbi.nlm.nih.gov/pubmed/?term=1970%09Heparin+therapy+in+septicemia+with+disseminated+intravascular+coagulation%09JAMES+J#%23) 1970;283(15):778-782.
6. Popov S , Kaufer C, Baymann E. Reactive fibrinolysis under Heparin treatment in haemorrhagical traumatic and septic shock. Langenbecks Archiv fur Chirurgie 1971;329:510.
7. [Wilson FE](http://www.ncbi.nlm.nih.gov/pubmed?term=Wilson FE%5BAuthor%5D&cauthor=true&cauthor_uid=4631175), [Morse SR](http://www.ncbi.nlm.nih.gov/pubmed?term=Morse SR%5BAuthor%5D&cauthor=true&cauthor_uid=4631175). Therapy of acute meningococcal infections: early volume expansion and prophylactic low dose heparin. [Am J Med Sci](http://www.ncbi.nlm.nih.gov/pubmed/?term=Am+J+Med+Sci.+1972+Dec%3B264(6)%3A445-55.Therapy+of+acute+meningococcal+infections%3A+early+volume+expansion+and#%23) 1972;264(6):445-455.
8. [Blum D](http://www.ncbi.nlm.nih.gov/pubmed?term=Blum D%5BAuthor%5D&cauthor=true&cauthor_uid=4766888), [Fondu P](http://www.ncbi.nlm.nih.gov/pubmed?term=Fondu P%5BAuthor%5D&cauthor=true&cauthor_uid=4766888), [Denolin-Reubens R](http://www.ncbi.nlm.nih.gov/pubmed?term=Denolin-Reubens R%5BAuthor%5D&cauthor=true&cauthor_uid=4766888), [Dubois J](http://www.ncbi.nlm.nih.gov/pubmed?term=Dubois J%5BAuthor%5D&cauthor=true&cauthor_uid=4766888). Early heparin therapy in 60 children with acute meningococcemia. Relationship between clinical manifestations and coagulation abnormalities. [Acta Chir Belg](http://www.ncbi.nlm.nih.gov/pubmed/?term=Acta+Chir+Belg.+1973+Jul%3B72(4)%3A288-97.Early+heparin+therapy+in+60+children+with+acute+meningococcemia.+Relationship#%23) 1973l;72(4):288-297.
9. [Hathaway WE](http://www.ncbi.nlm.nih.gov/pubmed?term=Hathaway WE%5BAuthor%5D&cauthor=true&cauthor_uid=4698969). Heparin therapy in acute meningococcemia. [J Pediatr](http://www.ncbi.nlm.nih.gov/pubmed/?term=Heparin+therapy+in+acute+meningococcemia.J+Pediatr.+1973+May%3B82(5)%3A900-1.#%23) 1973;82(5):900-901.
10. [Mitterstieler G](http://www.ncbi.nlm.nih.gov/pubmed?term=Mitterstieler G%5BAuthor%5D&cauthor=true&cauthor_uid=4698436), [Kurz R](http://www.ncbi.nlm.nih.gov/pubmed?term=Kurz R%5BAuthor%5D&cauthor=true&cauthor_uid=4698436), [Waltl H](http://www.ncbi.nlm.nih.gov/pubmed?term=Waltl H%5BAuthor%5D&cauthor=true&cauthor_uid=4698436), [Berger H](http://www.ncbi.nlm.nih.gov/pubmed?term=Berger H%5BAuthor%5D&cauthor=true&cauthor_uid=4698436). Fibrinolyte therapy of septicemic shock in infancy and childhood. [Padiatr Padol](http://www.ncbi.nlm.nih.gov/pubmed/?term=Padiatr+Padol.+1973%3B8(2)%3A225-31.%5BFibrinolyte+therapy+of+septicemic+shock+in+infancy+and+childhood%5D.#%23) 1973;8(2):225-231.
11. [de Aquino-Jaso ME](http://www.ncbi.nlm.nih.gov/pubmed?term=de Aquino-Jaso ME%5BAuthor%5D&cauthor=true&cauthor_uid=4433389), [Olvera-Hidalgo C](http://www.ncbi.nlm.nih.gov/pubmed?term=Olvera-Hidalgo C%5BAuthor%5D&cauthor=true&cauthor_uid=4433389), [Ramírez JI](http://www.ncbi.nlm.nih.gov/pubmed?term=Ramírez JI%5BAuthor%5D&cauthor=true&cauthor_uid=4433389). Importance of heparin in infants with consumption coagulation disease caused by septicemia. [Bol Med Hosp Infant Mex](http://www.ncbi.nlm.nih.gov/pubmed/?term=Importance+of+heparin+in+infants+with+consumption+coagulation+disease+caused+by+septicemiaBol+Med+Hosp+Infant+Mex.+1974+Jan-Feb%3B31(1)%3A73-84.#%23) 1974;31(1):73-84.
12. [Cavanagh D](http://www.ncbi.nlm.nih.gov/pubmed?term=Cavanagh D%5BAuthor%5D&cauthor=true&cauthor_uid=909855). Septic shock in a pregnant or recently pregnant woman. [Postgrad Med](http://www.ncbi.nlm.nih.gov/pubmed/?term=Septic+shock+in+a+pregnant+or+recently+pregnant+woman.Postgrad+Med.+1977+Oct%3B62(4)%3A62-8.#%23) 1977;62(4):62-68.
13. [Maszkiewicz W](http://www.ncbi.nlm.nih.gov/pubmed?term=Maszkiewicz W%5BAuthor%5D&cauthor=true&cauthor_uid=683758), [Czerchawski L](http://www.ncbi.nlm.nih.gov/pubmed?term=Czerchawski L%5BAuthor%5D&cauthor=true&cauthor_uid=683758), [Ornat K](http://www.ncbi.nlm.nih.gov/pubmed?term=Ornat K%5BAuthor%5D&cauthor=true&cauthor_uid=683758), [Manerowska D](http://www.ncbi.nlm.nih.gov/pubmed?term=Manerowska D%5BAuthor%5D&cauthor=true&cauthor_uid=683758). Long-term heparin treatment of thrombo-hemorrhagic complications of septicemia in a newborn infant. [Pediatr Pol](http://www.ncbi.nlm.nih.gov/pubmed/?term=Long-term+heparin+treatment+of+thrombo-hemorrhagic+complications+of+septicemiain+a+newborn+infantPediatr+Pol.+1978+May%3B53(5)%3A629-32.#%23) 1978;53(5):629-632.
14. [Schippan R](http://www.ncbi.nlm.nih.gov/pubmed?term=Schippan R%5BAuthor%5D&cauthor=true&cauthor_uid=661077). Septic shock. Special problems of disease and therapy in childhood [Kinderarztl Prax](http://www.ncbi.nlm.nih.gov/pubmed/?term=Septic+shock.+Special+problems+of+disease+and+therapy+in+childhoodKinderarztl+Prax.+1978+Apr%3B46(4)%3A203-10.#%23) 1978;46(4):203-210.
15. [Iturriaga Ruiz S](http://www.ncbi.nlm.nih.gov/pubmed?term=Iturriaga Ruiz S%5BAuthor%5D&cauthor=true&cauthor_uid=550213), [Klein Torres F](http://www.ncbi.nlm.nih.gov/pubmed?term=Klein Torres F%5BAuthor%5D&cauthor=true&cauthor_uid=550213), [Silva Lake O](http://www.ncbi.nlm.nih.gov/pubmed?term=Silva Lake O%5BAuthor%5D&cauthor=true&cauthor_uid=550213), [Lattus Olmos J](http://www.ncbi.nlm.nih.gov/pubmed?term=Lattus Olmos J%5BAuthor%5D&cauthor=true&cauthor_uid=550213), [Tisné Brousse L](http://www.ncbi.nlm.nih.gov/pubmed?term=Tisné Brousse L%5BAuthor%5D&cauthor=true&cauthor_uid=550213). Reduction of maternal mortality due to post-abortion septic shock, by medical and surgical treatment. [Rev Chil Obstet Ginecol](http://www.ncbi.nlm.nih.gov/pubmed/?term=Reduction+of+maternal+mortality+due+to+post-abortion+septic+shock%2C+by+medicaland+surgical+treatmentRev+Chil+Obstet+Ginecol.+1978%3B43(6)%3A330-43.#%23) 1978;43(6):330-343.
16. [Wytrychowski M](http://www.ncbi.nlm.nih.gov/pubmed?term=Wytrychowski M%5BAuthor%5D&cauthor=true&cauthor_uid=390515), [Czerchawski L](http://www.ncbi.nlm.nih.gov/pubmed?term=Czerchawski L%5BAuthor%5D&cauthor=true&cauthor_uid=390515), [Morawska Z](http://www.ncbi.nlm.nih.gov/pubmed?term=Morawska Z%5BAuthor%5D&cauthor=true&cauthor_uid=390515), [Kibler M](http://www.ncbi.nlm.nih.gov/pubmed?term=Kibler M%5BAuthor%5D&cauthor=true&cauthor_uid=390515). Unsuccessful heparin treatment of intravascular dissemination coagulation in infants with E. coli septicemia. [Pol Tyg Lek](http://www.ncbi.nlm.nih.gov/pubmed/?term=Unsuccessful+heparin+treatment+of+intravascular+dissemination+coagulation+ininfants+with+E.+coli+septicemiaPol+Tyg+Lek.+1979+Nov+5%3B34(45)%3A1755-7.#%23) 1979;34(45):1755-1757.
17. Abizanda Campos R, Valle Herráez FX, Guiscafré Amer J, Jordá Marcos R, Claramonte Porcar R. Study of 51 cases of disseminated intravascular coagulation with reference to the association with acute renal failure. Results in severely ill patients (author's transl) Med Clin (Barc) 1981;76(6):262-266.
18. [Chaĭtsev VG](http://www.ncbi.nlm.nih.gov/pubmed?term=Chaĭtsev VG%5BAuthor%5D&cauthor=true&cauthor_uid=7063939), [Galushkin IP](http://www.ncbi.nlm.nih.gov/pubmed?term=Galushkin IP%5BAuthor%5D&cauthor=true&cauthor_uid=7063939), [Putintseva OV](http://www.ncbi.nlm.nih.gov/pubmed?term=Putintseva OV%5BAuthor%5D&cauthor=true&cauthor_uid=7063939), [Lezhenina TM](http://www.ncbi.nlm.nih.gov/pubmed?term=Lezhenina TM%5BAuthor%5D&cauthor=true&cauthor_uid=7063939). Use of heparin in severe infectious diseases. [Sov Med](http://www.ncbi.nlm.nih.gov/pubmed/?term=Use+of+heparin+in+severe+infectious+diseasesSov+Med.+1982%3B(1)%3A35-9.#%23) 1982;(1):35-39.
19. [Grygalewicz J](http://www.ncbi.nlm.nih.gov/pubmed?term=Grygalewicz J%5BAuthor%5D&cauthor=true&cauthor_uid=7182777), [Niznikowska-Marks MJ](http://www.ncbi.nlm.nih.gov/pubmed?term=Niznikowska-Marks MJ%5BAuthor%5D&cauthor=true&cauthor_uid=7182777), [Kozierowska T](http://www.ncbi.nlm.nih.gov/pubmed?term=Kozierowska T%5BAuthor%5D&cauthor=true&cauthor_uid=7182777), [Rzacka D](http://www.ncbi.nlm.nih.gov/pubmed?term=Rzacka D%5BAuthor%5D&cauthor=true&cauthor_uid=7182777), [Janus M](http://www.ncbi.nlm.nih.gov/pubmed?term=Janus M%5BAuthor%5D&cauthor=true&cauthor_uid=7182777). Use of subcutaneous heparin in children with severe infection. Prevention of coagulopathy or additional method of treatment? [Pediatr Pol.](http://www.ncbi.nlm.nih.gov/pubmed/?term=Use+of+subcutaneous+heparin+in+children+with+severe+infection.+Prevention+ofcoagulopathy+or+additional+method+of+treatment%3FPediatr+Pol.+1982+Dec%3B57(12)%3A1003-13.#%23) 1982;57(12):1003-1013.
20. [Taenaka N](http://www.ncbi.nlm.nih.gov/pubmed?term=Taenaka N%5BAuthor%5D&cauthor=true&cauthor_uid=6411432), [Shimada Y](http://www.ncbi.nlm.nih.gov/pubmed?term=Shimada Y%5BAuthor%5D&cauthor=true&cauthor_uid=6411432), [Hirata T](http://www.ncbi.nlm.nih.gov/pubmed?term=Hirata T%5BAuthor%5D&cauthor=true&cauthor_uid=6411432), [Nishijima MK](http://www.ncbi.nlm.nih.gov/pubmed?term=Nishijima MK%5BAuthor%5D&cauthor=true&cauthor_uid=6411432), [Takezawa J](http://www.ncbi.nlm.nih.gov/pubmed?term=Takezawa J%5BAuthor%5D&cauthor=true&cauthor_uid=6411432), [Yoshiya I](http://www.ncbi.nlm.nih.gov/pubmed?term=Yoshiya I%5BAuthor%5D&cauthor=true&cauthor_uid=6411432), [Kambayashi J](http://www.ncbi.nlm.nih.gov/pubmed?term=Kambayashi J%5BAuthor%5D&cauthor=true&cauthor_uid=6411432). Gabexate mesilate (FOY) therapy of disseminated intravascular coagulation due to sepsis. [Crit Care Med](http://www.ncbi.nlm.nih.gov/pubmed/?term=1983%09Gabexate+mesilate+(FOY)+therapy+of+disseminated+intravascular+coagulation+due+to+sepsis%09NOBUYUKI+TAENAKA.+MD#%23) 1983;11(9):735-738.
21. [Lytkin MI](http://www.ncbi.nlm.nih.gov/pubmed?term=Lytkin MI%5BAuthor%5D&cauthor=true&cauthor_uid=6623822), [Kostiuchenko AL](http://www.ncbi.nlm.nih.gov/pubmed?term=Kostiuchenko AL%5BAuthor%5D&cauthor=true&cauthor_uid=6623822). Major trends in the treatment of patients with septic shock [Vestn Khir Im I I Grek](http://www.ncbi.nlm.nih.gov/pubmed/?term=Major+trends+in+the+treatment+of+patients+with+septic+shockVestn+Khir+Im+I+I+Grek.+1983+Jul%3B131(7)%3A3-10.#%23) 1983l;131(7):3-10.
22. [Haneberg B](http://www.ncbi.nlm.nih.gov/pubmed?term=Haneberg B%5BAuthor%5D&cauthor=true&cauthor_uid=6353278), [Gutteberg TJ](http://www.ncbi.nlm.nih.gov/pubmed?term=Gutteberg TJ%5BAuthor%5D&cauthor=true&cauthor_uid=6353278), [Moe PJ](http://www.ncbi.nlm.nih.gov/pubmed?term=Moe PJ%5BAuthor%5D&cauthor=true&cauthor_uid=6353278), [Osterud B](http://www.ncbi.nlm.nih.gov/pubmed?term=Osterud B%5BAuthor%5D&cauthor=true&cauthor_uid=6353278), [Bjorvatn B](http://www.ncbi.nlm.nih.gov/pubmed?term=Bjorvatn B%5BAuthor%5D&cauthor=true&cauthor_uid=6353278), [Lehmann EH](http://www.ncbi.nlm.nih.gov/pubmed?term=Lehmann EH%5BAuthor%5D&cauthor=true&cauthor_uid=6353278). Heparin for infants and children with meningococcal septicemia. Results of a randomized therapeutic trial. [NIPH Ann](http://www.ncbi.nlm.nih.gov/pubmed/?term=Heparin+for+infants+and+children+with+meningococcal+septicemia.+Results+of+arandomized+therapeutic+trialNIPH+Ann.+1983+Jun%3B6(1)%3A43-7.#%23) 1983;6(1):43-47.
23. [Godula-Stuglik U](http://www.ncbi.nlm.nih.gov/pubmed?term=Godula-Stuglik U%5BAuthor%5D&cauthor=true&cauthor_uid=6634257). Results of treatment of disseminated intravascular coagulation in newborn infants with septicemia [Pediatr Pol](http://www.ncbi.nlm.nih.gov/pubmed/?term=Results+of+treatment+of+disseminated+intravascular+coagulation+in+newborninfants+with+septicemiaPediatr+Pol.+1983+May%3B58(5)%3A425-35.#%23) 1983;58(5):425-435.
24. [Dobrovol'skiĭ VI](http://www.ncbi.nlm.nih.gov/pubmed?term=Dobrovol'skiĭ VI%5BAuthor%5D&cauthor=true&cauthor_uid=6463755), [Makatsariia AD](http://www.ncbi.nlm.nih.gov/pubmed?term=Makatsariia AD%5BAuthor%5D&cauthor=true&cauthor_uid=6463755). Heparin therapy in bacterial shock and acute kidney failure. [Sov Med](http://www.ncbi.nlm.nih.gov/pubmed/?term=Heparin+therapy+in+bacterial+shock+and+acute+kidney+failureSov+Med.+1984%3B(6)%3A90-3.#%23) 1984;(6):90-93.
25. [Smith OP](http://www.ncbi.nlm.nih.gov/pubmed?term=Smith OP%5BAuthor%5D&cauthor=true&cauthor_uid=9393338), [White B](http://www.ncbi.nlm.nih.gov/pubmed?term=White B%5BAuthor%5D&cauthor=true&cauthor_uid=9393338), [Vaughan D](http://www.ncbi.nlm.nih.gov/pubmed?term=Vaughan D%5BAuthor%5D&cauthor=true&cauthor_uid=9393338), [Rafferty M](http://www.ncbi.nlm.nih.gov/pubmed?term=Rafferty M%5BAuthor%5D&cauthor=true&cauthor_uid=9393338), [Claffey L](http://www.ncbi.nlm.nih.gov/pubmed?term=Claffey L%5BAuthor%5D&cauthor=true&cauthor_uid=9393338), [Lyons B](http://www.ncbi.nlm.nih.gov/pubmed?term=Lyons B%5BAuthor%5D&cauthor=true&cauthor_uid=9393338), [Casey W](http://www.ncbi.nlm.nih.gov/pubmed?term=Casey W%5BAuthor%5D&cauthor=true&cauthor_uid=9393338). Use of protein-C concentrate, heparin, and haemodiafiltration in meningococcus-induced purpura fulminans. [Lancet](http://www.ncbi.nlm.nih.gov/pubmed/?term=1997%09Use+of+protein-C+concentrate%2C+heparin%2C+and+haemodiafiltration+in+meningococcus-induced+purpura+fulminans%09O+P+Smith#%23) 1997;350(9091):1590-1593.
26. Ghanem J , San Juan J , Enrico E , Prieto R , Villar O , Perelmutter H, Scaziotta A. A collaborative study on the treatment of coagulation disturbances with antithrombin III or heparin. Thrombosis and Haemostasis 1997;Suppl, 517
27. [Hofmann](http://cnplinker.cnpeak.com/outline_article.jsp?channelid=70050&searchword=authorname%3D(M. Hofmann)) M, [Rest A,](http://cnplinker.cnpeak.com/outline_article.jsp?channelid=70050&searchword=authorname%3D(A. Rest)) [Hafner](http://cnplinker.cnpeak.com/outline_article.jsp?channelid=70050&searchword=authorname%3D(G. Hafner)) G, [Tanner](http://cnplinker.cnpeak.com/outline_article.jsp?channelid=70050&searchword=authorname%3D(B. Tanner)) B, [Brockerhoff](http://cnplinker.cnpeak.com/outline_article.jsp?channelid=70050&searchword=authorname%3D(P. Brockerhoff)) P, [Weilemann](http://cnplinker.cnpeak.com/outline_article.jsp?channelid=70050&searchword=authorname%3D(L. S. Weilemann)) LS. Evaluation of coagulation and fibrinolytics parameters in the therapeutic management of disseminated intravascular coagulation with sepsis by low molecular weight/heparin. [Der Anaesthesist](http://cnplinker.cnpeak.com/outline_issue.jsp?prechannelid=70050&channelid=70040&searchword=jid%3D1099&journaltitle=Der+Anaesthesist&issn=0003-2417) 1997;8:689-696.
28. [Boldt J](http://www.ncbi.nlm.nih.gov/pubmed?term=Boldt J%5BAuthor%5D&cauthor=true&cauthor_uid=9921711), [Papsdorf M](http://www.ncbi.nlm.nih.gov/pubmed?term=Papsdorf M%5BAuthor%5D&cauthor=true&cauthor_uid=9921711), [Piper SN](http://www.ncbi.nlm.nih.gov/pubmed?term=Piper SN%5BAuthor%5D&cauthor=true&cauthor_uid=9921711), [Rothe A](http://www.ncbi.nlm.nih.gov/pubmed?term=Rothe A%5BAuthor%5D&cauthor=true&cauthor_uid=9921711), [Hempelmann G](http://www.ncbi.nlm.nih.gov/pubmed?term=Hempelmann G%5BAuthor%5D&cauthor=true&cauthor_uid=9921711). Continuous heparinization and circulating adhesion molecules in the critically ill. [Shock](http://www.ncbi.nlm.nih.gov/pubmed/?term=1999%09Continuous+heparinization+and+circulating+adhesion+molecules+in+the+critically+ill%09JoachimBoldt#%23) 1999;11(1):13-18.
29. [Rublee D](http://www.ncbi.nlm.nih.gov/pubmed?term=Rublee D%5BAuthor%5D&cauthor=true&cauthor_uid=12225612), [Opal SM](http://www.ncbi.nlm.nih.gov/pubmed?term=Opal SM%5BAuthor%5D&cauthor=true&cauthor_uid=12225612), [Schramm W](http://www.ncbi.nlm.nih.gov/pubmed?term=Schramm W%5BAuthor%5D&cauthor=true&cauthor_uid=12225612), [Keinecke HO](http://www.ncbi.nlm.nih.gov/pubmed?term=Keinecke HO%5BAuthor%5D&cauthor=true&cauthor_uid=12225612), [Knaub S](http://www.ncbi.nlm.nih.gov/pubmed?term=Knaub S%5BAuthor%5D&cauthor=true&cauthor_uid=12225612). Quality of life effects of antithrombin III in sepsis survivors: results from the KyberSept trial [ISRCTN22931023 [Crit Care.](http://www.ncbi.nlm.nih.gov/pubmed/?term=2002%09Quality+of+life+effects+of+antithrombin+III+in+sepsis+survivors+results+from+the+KyberSept+trial%09Dale+Rublee#%23) 2002;6(4):349-356.
30. [Messori A](http://www.ncbi.nlm.nih.gov/pubmed?term=Messori A%5BAuthor%5D&cauthor=true&cauthor_uid=12398786), [Vacca F](http://www.ncbi.nlm.nih.gov/pubmed?term=Vacca F%5BAuthor%5D&cauthor=true&cauthor_uid=12398786), [Vaiani M](http://www.ncbi.nlm.nih.gov/pubmed?term=Vaiani M%5BAuthor%5D&cauthor=true&cauthor_uid=12398786), [Trippoli S](http://www.ncbi.nlm.nih.gov/pubmed?term=Trippoli S%5BAuthor%5D&cauthor=true&cauthor_uid=12398786), [Gruppo di Studio sull'antitrombina III](http://www.ncbi.nlm.nih.gov/pubmed?term=Gruppo di Studio sull'antitrombina III%5BCorporate Author%5D). Antithrombin III in patients admitted to intensive care units: a multicenter observational study. [Crit Care](http://www.ncbi.nlm.nih.gov/pubmed/?term=2002%09Antithrombin+III+in+patients+admitted+to+intensive+care+units+a+multicenter+observational+study%09Andrea+Messori#%23) 2002;6(5):447-451.
31. [Davidson BL](http://www.ncbi.nlm.nih.gov/pubmed?term=Davidson BL%5BAuthor%5D&cauthor=true&cauthor_uid=12324565), [Geerts WH](http://www.ncbi.nlm.nih.gov/pubmed?term=Geerts WH%5BAuthor%5D&cauthor=true&cauthor_uid=12324565), [Lensing AW](http://www.ncbi.nlm.nih.gov/pubmed?term=Lensing AW%5BAuthor%5D&cauthor=true&cauthor_uid=12324565). Low-dose heparin for severe sepsis. [N Engl J Med](http://www.ncbi.nlm.nih.gov/pubmed/12324565#%23) 2002;347(13):1036-1037.
32. [Derhaschnig U](http://www.ncbi.nlm.nih.gov/pubmed?term=Derhaschnig U%5BAuthor%5D&cauthor=true&cauthor_uid=12682480), [Pernerstorfer T](http://www.ncbi.nlm.nih.gov/pubmed?term=Pernerstorfer T%5BAuthor%5D&cauthor=true&cauthor_uid=12682480), [Knechtelsdorfer M](http://www.ncbi.nlm.nih.gov/pubmed?term=Knechtelsdorfer M%5BAuthor%5D&cauthor=true&cauthor_uid=12682480), [Hollenstein U](http://www.ncbi.nlm.nih.gov/pubmed?term=Hollenstein U%5BAuthor%5D&cauthor=true&cauthor_uid=12682480), [Panzer S](http://www.ncbi.nlm.nih.gov/pubmed?term=Panzer S%5BAuthor%5D&cauthor=true&cauthor_uid=12682480), [Jilma B](http://www.ncbi.nlm.nih.gov/pubmed?term=Jilma B%5BAuthor%5D&cauthor=true&cauthor_uid=12682480). Evaluation of antiinflammatory and antiadhesive effects of heparins in human endotoxemia. [Crit Care Med](http://www.ncbi.nlm.nih.gov/pubmed/?term=2003%09Evaluation+of+antiinflammatory+and+antiadhesive+effects+of+heparins+in+human+endotoxemia%09Ulla+Derhaschnig%2C+MD#%23) 2003;31(4):1108-1112.
33. [Koestenberger M](http://www.ncbi.nlm.nih.gov/pubmed?term=Koestenberger M%5BAuthor%5D&cauthor=true&cauthor_uid=16188810), [Cvirn G](http://www.ncbi.nlm.nih.gov/pubmed?term=Cvirn G%5BAuthor%5D&cauthor=true&cauthor_uid=16188810), [Gallistl S](http://www.ncbi.nlm.nih.gov/pubmed?term=Gallistl S%5BAuthor%5D&cauthor=true&cauthor_uid=16188810), [Kutschera J](http://www.ncbi.nlm.nih.gov/pubmed?term=Kutschera J%5BAuthor%5D&cauthor=true&cauthor_uid=16188810), [Muntean W](http://www.ncbi.nlm.nih.gov/pubmed?term=Muntean W%5BAuthor%5D&cauthor=true&cauthor_uid=16188810). Recombinant human activated protein C, heparin and melagatran in umbilical cord versus adult plasma. [Acta Paediatr](http://www.ncbi.nlm.nih.gov/pubmed/?term=2005%09Recombinant+human+activated+protein+C%2C+heparin+and+melagatran+in+umbilical+cord+versus+adult+plasm%09MARTIN+KOESTENBERGER#%23) 2005;94(7):884-889.
34. [Agarwal R](http://www.ncbi.nlm.nih.gov/pubmed?term=Agarwal R%5BAuthor%5D&cauthor=true&cauthor_uid=16007415), [Gupta D](http://www.ncbi.nlm.nih.gov/pubmed?term=Gupta D%5BAuthor%5D&cauthor=true&cauthor_uid=16007415). Anticoagulation in sepsis: is low-dose heparin as effective as activated protein C? [Intensive Care Med](http://www.ncbi.nlm.nih.gov/pubmed/?term=Anticoagulation+in+sepsis%3A+Is+low-dose+heparin+as+effective+as+activated+protein+C%3F#%23) 2005;31(9):1297–1298.
35. [Gullo A](http://www.ncbi.nlm.nih.gov/pubmed?term=Gullo A%5BAuthor%5D&cauthor=true&cauthor_uid=16288186), [Iscra F](http://www.ncbi.nlm.nih.gov/pubmed?term=Iscra F%5BAuthor%5D&cauthor=true&cauthor_uid=16288186), [Di Capua G](http://www.ncbi.nlm.nih.gov/pubmed?term=Di Capua G%5BAuthor%5D&cauthor=true&cauthor_uid=16288186), [Berlot G](http://www.ncbi.nlm.nih.gov/pubmed?term=Berlot G%5BAuthor%5D&cauthor=true&cauthor_uid=16288186), [Lucangelo U](http://www.ncbi.nlm.nih.gov/pubmed?term=Lucangelo U%5BAuthor%5D&cauthor=true&cauthor_uid=16288186), [Peratoner A](http://www.ncbi.nlm.nih.gov/pubmed?term=Peratoner A%5BAuthor%5D&cauthor=true&cauthor_uid=16288186), [Fasiolo S](http://www.ncbi.nlm.nih.gov/pubmed?term=Fasiolo S%5BAuthor%5D&cauthor=true&cauthor_uid=16288186), [Viviani M](http://www.ncbi.nlm.nih.gov/pubmed?term=Viviani M%5BAuthor%5D&cauthor=true&cauthor_uid=16288186), [Consales C](http://www.ncbi.nlm.nih.gov/pubmed?term=Consales C%5BAuthor%5D&cauthor=true&cauthor_uid=16288186), [Zicari A](http://www.ncbi.nlm.nih.gov/pubmed?term=Zicari A%5BAuthor%5D&cauthor=true&cauthor_uid=16288186). Systemic and organ dysfunction response during infusion of recombinant human activated protein C (rhAPC) in severe sepsis and septic shock. [Minerva Anestesiol](http://www.ncbi.nlm.nih.gov/pubmed/16288186#%23) 2005;71(12):785-801.
36. [Dhainaut JF](http://www.ncbi.nlm.nih.gov/pubmed?term=Dhainaut JF%5BAuthor%5D&cauthor=true&cauthor_uid=15699837), [Shorr AF](http://www.ncbi.nlm.nih.gov/pubmed?term=Shorr AF%5BAuthor%5D&cauthor=true&cauthor_uid=15699837), [Macias WL](http://www.ncbi.nlm.nih.gov/pubmed?term=Macias WL%5BAuthor%5D&cauthor=true&cauthor_uid=15699837), [Kollef MJ](http://www.ncbi.nlm.nih.gov/pubmed?term=Kollef MJ%5BAuthor%5D&cauthor=true&cauthor_uid=15699837), [Levi M](http://www.ncbi.nlm.nih.gov/pubmed?term=Levi M%5BAuthor%5D&cauthor=true&cauthor_uid=15699837), [Reinhart K](http://www.ncbi.nlm.nih.gov/pubmed?term=Reinhart K%5BAuthor%5D&cauthor=true&cauthor_uid=15699837), [Nelson DR](http://www.ncbi.nlm.nih.gov/pubmed?term=Nelson DR%5BAuthor%5D&cauthor=true&cauthor_uid=15699837). Dynamic evolution of coagulopathy in the first day of severe sepsis: Relationship with mortality and organ failure. Crit Care Med 2005;33:341–348.
37. [Jaimes F](http://www.ncbi.nlm.nih.gov/pubmed?term=Jaimes F%5BAuthor%5D&cauthor=true&cauthor_uid=16729879), [De la Rosa G](http://www.ncbi.nlm.nih.gov/pubmed?term=De la Rosa G%5BAuthor%5D&cauthor=true&cauthor_uid=16729879), [Arango C](http://www.ncbi.nlm.nih.gov/pubmed?term=Arango C%5BAuthor%5D&cauthor=true&cauthor_uid=16729879), [Fortich F](http://www.ncbi.nlm.nih.gov/pubmed?term=Fortich F%5BAuthor%5D&cauthor=true&cauthor_uid=16729879), [Morales C](http://www.ncbi.nlm.nih.gov/pubmed?term=Morales C%5BAuthor%5D&cauthor=true&cauthor_uid=16729879), [Aguirre D](http://www.ncbi.nlm.nih.gov/pubmed?term=Aguirre D%5BAuthor%5D&cauthor=true&cauthor_uid=16729879), [Patiño P](http://www.ncbi.nlm.nih.gov/pubmed?term=Patiño P%5BAuthor%5D&cauthor=true&cauthor_uid=16729879). A randomized clinical trial of unfractioned heparin for treatment of sepsis (the HETRASE study): design and rationale [NCT00100308]. [Trials](http://www.ncbi.nlm.nih.gov/pubmed/?term=2006%09A+randomized+clinical+trial+of+unfractioned+heparin+for+treatment+of+sepsis%09Fabián+Jaimes#%23) 2006;7:19.
38. [Hoffmann JN](http://www.ncbi.nlm.nih.gov/pubmed?term=Hoffmann JN%5BAuthor%5D&cauthor=true&cauthor_uid=16676077), [Wiedermann CJ](http://www.ncbi.nlm.nih.gov/pubmed?term=Wiedermann CJ%5BAuthor%5D&cauthor=true&cauthor_uid=16676077), [Juers M](http://www.ncbi.nlm.nih.gov/pubmed?term=Juers M%5BAuthor%5D&cauthor=true&cauthor_uid=16676077), [Ostermann H](http://www.ncbi.nlm.nih.gov/pubmed?term=Ostermann H%5BAuthor%5D&cauthor=true&cauthor_uid=16676077), [Kienast J](http://www.ncbi.nlm.nih.gov/pubmed?term=Kienast J%5BAuthor%5D&cauthor=true&cauthor_uid=16676077), [Briegel J](http://www.ncbi.nlm.nih.gov/pubmed?term=Briegel J%5BAuthor%5D&cauthor=true&cauthor_uid=16676077), [Strauss R](http://www.ncbi.nlm.nih.gov/pubmed?term=Strauss R%5BAuthor%5D&cauthor=true&cauthor_uid=16676077), [Warren BL](http://www.ncbi.nlm.nih.gov/pubmed?term=Warren BL%5BAuthor%5D&cauthor=true&cauthor_uid=16676077), [Opal SM](http://www.ncbi.nlm.nih.gov/pubmed?term=Opal SM%5BAuthor%5D&cauthor=true&cauthor_uid=16676077); [KyberSept investigators](http://www.ncbi.nlm.nih.gov/pubmed?term=KyberSept investigators%5BCorporate Author%5D). Benefit/risk profile of high-dose antithrombin in patients with severe sepsis treated with and without concomitant heparin. [Thromb Haemost](http://www.ncbi.nlm.nih.gov/pubmed/?term=2006%09Benefit+risk+profile+of+high-dose+antithrombin+in+patients+with+severe+sepsis+treated+with+and+without+concomitant+heparin%09Johannes+N#%23) 2006;95(5):850-856.
39. [Kirschenbaum LA](http://www.ncbi.nlm.nih.gov/pubmed?term=Kirschenbaum LA%5BAuthor%5D&cauthor=true&cauthor_uid=16775572), [Lopez WC](http://www.ncbi.nlm.nih.gov/pubmed?term=Lopez WC%5BAuthor%5D&cauthor=true&cauthor_uid=16775572), [Ohrum P](http://www.ncbi.nlm.nih.gov/pubmed?term=Ohrum P%5BAuthor%5D&cauthor=true&cauthor_uid=16775572), [Tsen A](http://www.ncbi.nlm.nih.gov/pubmed?term=Tsen A%5BAuthor%5D&cauthor=true&cauthor_uid=16775572), [Khazin J](http://www.ncbi.nlm.nih.gov/pubmed?term=Khazin J%5BAuthor%5D&cauthor=true&cauthor_uid=16775572), [Astiz ME](http://www.ncbi.nlm.nih.gov/pubmed?term=Astiz ME%5BAuthor%5D&cauthor=true&cauthor_uid=16775572). Effect of recombinant activated protein C and low-dose heparin on neutrophil-endothelial cell interactions in septic shock. [Crit Care Med](http://www.ncbi.nlm.nih.gov/pubmed/?term=Effect+of+recombinant+activated+protein+C+and+low-dose+heparin+on+neutrophil-endothelial+cell+interactions+in+septic+shock) 2006; 34(8):2207–2212.
40. [Wiedermann CJ](http://www.ncbi.nlm.nih.gov/pubmed?term=Wiedermann CJ%5BAuthor%5D&cauthor=true&cauthor_uid=16424704), [Hoffmann JN](http://www.ncbi.nlm.nih.gov/pubmed?term=Hoffmann JN%5BAuthor%5D&cauthor=true&cauthor_uid=16424704), [Juers M](http://www.ncbi.nlm.nih.gov/pubmed?term=Juers M%5BAuthor%5D&cauthor=true&cauthor_uid=16424704), [Ostermann H](http://www.ncbi.nlm.nih.gov/pubmed?term=Ostermann H%5BAuthor%5D&cauthor=true&cauthor_uid=16424704), [Kienast J](http://www.ncbi.nlm.nih.gov/pubmed?term=Kienast J%5BAuthor%5D&cauthor=true&cauthor_uid=16424704), [Briegel J](http://www.ncbi.nlm.nih.gov/pubmed?term=Briegel J%5BAuthor%5D&cauthor=true&cauthor_uid=16424704), [Strauss R](http://www.ncbi.nlm.nih.gov/pubmed?term=Strauss R%5BAuthor%5D&cauthor=true&cauthor_uid=16424704), [Keinecke HO](http://www.ncbi.nlm.nih.gov/pubmed?term=Keinecke HO%5BAuthor%5D&cauthor=true&cauthor_uid=16424704), [Warren BL](http://www.ncbi.nlm.nih.gov/pubmed?term=Warren BL%5BAuthor%5D&cauthor=true&cauthor_uid=16424704), [Opal SM](http://www.ncbi.nlm.nih.gov/pubmed?term=Opal SM%5BAuthor%5D&cauthor=true&cauthor_uid=16424704); [KyberSept Investigators](http://www.ncbi.nlm.nih.gov/pubmed?term=KyberSept Investigators%5BCorporate Author%5D). High-dose antithrombin III in the treatment of severe sepsis in patients with a high risk of death: efficacy and safety. [Crit Care Med](http://www.ncbi.nlm.nih.gov/pubmed/?term=2006%09High-dose+antithrombin+III+in+the+treatment+of+severe+sepsis+in+patients+with+a+high+risk+of+death+Efficacy+and+safety%09Christian+J.#%23) 2006;34(2):285-292.
41. [Kienast J](http://www.ncbi.nlm.nih.gov/pubmed?term=Kienast J%5BAuthor%5D&cauthor=true&cauthor_uid=16409457), [Juers M](http://www.ncbi.nlm.nih.gov/pubmed?term=Juers M%5BAuthor%5D&cauthor=true&cauthor_uid=16409457), [Wiedermann CJ](http://www.ncbi.nlm.nih.gov/pubmed?term=Wiedermann CJ%5BAuthor%5D&cauthor=true&cauthor_uid=16409457), [Hoffmann JN](http://www.ncbi.nlm.nih.gov/pubmed?term=Hoffmann JN%5BAuthor%5D&cauthor=true&cauthor_uid=16409457), [Ostermann H](http://www.ncbi.nlm.nih.gov/pubmed?term=Ostermann H%5BAuthor%5D&cauthor=true&cauthor_uid=16409457), [Strauss R](http://www.ncbi.nlm.nih.gov/pubmed?term=Strauss R%5BAuthor%5D&cauthor=true&cauthor_uid=16409457), [Keinecke HO](http://www.ncbi.nlm.nih.gov/pubmed?term=Keinecke HO%5BAuthor%5D&cauthor=true&cauthor_uid=16409457), [Warren BL](http://www.ncbi.nlm.nih.gov/pubmed?term=Warren BL%5BAuthor%5D&cauthor=true&cauthor_uid=16409457), [Opal SM](http://www.ncbi.nlm.nih.gov/pubmed?term=Opal SM%5BAuthor%5D&cauthor=true&cauthor_uid=16409457); [KyberSept investigators](http://www.ncbi.nlm.nih.gov/pubmed?term=KyberSept investigators%5BCorporate Author%5D). Treatment effects of high-dose antithrombin without concomitant heparin in patients with severe sepsis with or without disseminated intravascular coagulation. [J Thromb Haemost](http://www.ncbi.nlm.nih.gov/pubmed/16409457#%23) 2006;4(1):90-97.
42. Zarychanski R, Gulati H, Roberts D, Sharma S, Houston DS, Kumar A. Systemically administered therapeutic dose heparin is associated with increased survival in patients diagnosed with septic shock. 36th Critical Care Congress of the Society-of-Critical-Care-Medicine CY FEB 18-21, 2007.
43. Dries D, Awad S, Woodward B, Wang D, Williams M. Surgical patients from the XPRESS study: prophylactic heparin in severe sepsis patients receiving drotrecogin alfa(activated). Abstracts of the 36th Critical Care Congress, Orlando, Florida, USA, February 17-21, 2007. [Crit Care Med](http://www.ncbi.nlm.nih.gov/pubmed/?term=2007%09Abstracts+of+the+36th+Critical+Care+Congress%2C+Orlando%2C+Florida%2C+USA%2C+February+17-21%2C+2007%09John+S+Giuliano#%23) 2006;34(12) (Abstr Suppl):A1-176.
44. [Saito H](http://www.ncbi.nlm.nih.gov/pubmed?term=Saito H%5BAuthor%5D&cauthor=true&cauthor_uid=17059423), [Maruyama I](http://www.ncbi.nlm.nih.gov/pubmed?term=Maruyama I%5BAuthor%5D&cauthor=true&cauthor_uid=17059423), [Shimazaki S](http://www.ncbi.nlm.nih.gov/pubmed?term=Shimazaki S%5BAuthor%5D&cauthor=true&cauthor_uid=17059423), [Yamamoto Y](http://www.ncbi.nlm.nih.gov/pubmed?term=Yamamoto Y%5BAuthor%5D&cauthor=true&cauthor_uid=17059423), [Aikawa N](http://www.ncbi.nlm.nih.gov/pubmed?term=Aikawa N%5BAuthor%5D&cauthor=true&cauthor_uid=17059423), [Ohno R](http://www.ncbi.nlm.nih.gov/pubmed?term=Ohno R%5BAuthor%5D&cauthor=true&cauthor_uid=17059423), [Hirayama A](http://www.ncbi.nlm.nih.gov/pubmed?term=Hirayama A%5BAuthor%5D&cauthor=true&cauthor_uid=17059423), [Matsuda T](http://www.ncbi.nlm.nih.gov/pubmed?term=Matsuda T%5BAuthor%5D&cauthor=true&cauthor_uid=17059423), [Asakura H](http://www.ncbi.nlm.nih.gov/pubmed?term=Asakura H%5BAuthor%5D&cauthor=true&cauthor_uid=17059423), [Nakashima M](http://www.ncbi.nlm.nih.gov/pubmed?term=Nakashima M%5BAuthor%5D&cauthor=true&cauthor_uid=17059423), et al. Efficacy and safety of recombinant human soluble thrombomodulin (ART-123) in disseminated intravascular coagulation: results of a phase III, randomized, double-blind clinical trial. [J Thromb Haemost.](http://www.ncbi.nlm.nih.gov/pubmed/?term=2007%09Efficacy+and+safety+of+recombinant+human+soluble+thrombomodulin+(ART-123)+in+disseminated+intravascular+coagulation+results+of+a+phase+III%2C+randomized%2C+double-blind+clinical+trial%09H.+SAITO#%23) 2007;5(1):31-41.
45. [Pechlaner C](http://www.ncbi.nlm.nih.gov/pubmed?term=Pechlaner C%5BAuthor%5D&cauthor=true&cauthor_uid=17765516), [Joannidis M](http://www.ncbi.nlm.nih.gov/pubmed?term=Joannidis M%5BAuthor%5D&cauthor=true&cauthor_uid=17765516). Heparin in sepsis treatment. [Lancet](http://www.ncbi.nlm.nih.gov/pubmed/17765516#%23) 2007;370(9589):738; author reply 738.
46. [Levi M](http://www.ncbi.nlm.nih.gov/pubmed?term=Levi M%5BAuthor%5D&cauthor=true&cauthor_uid=17556722), [Levy M](http://www.ncbi.nlm.nih.gov/pubmed?term=Levy M%5BAuthor%5D&cauthor=true&cauthor_uid=17556722), [Williams MD](http://www.ncbi.nlm.nih.gov/pubmed?term=Williams MD%5BAuthor%5D&cauthor=true&cauthor_uid=17556722), [Douglas I](http://www.ncbi.nlm.nih.gov/pubmed?term=Douglas I%5BAuthor%5D&cauthor=true&cauthor_uid=17556722), [Artigas A](http://www.ncbi.nlm.nih.gov/pubmed?term=Artigas A%5BAuthor%5D&cauthor=true&cauthor_uid=17556722), [Antonelli M](http://www.ncbi.nlm.nih.gov/pubmed?term=Antonelli M%5BAuthor%5D&cauthor=true&cauthor_uid=17556722), [Wyncoll D](http://www.ncbi.nlm.nih.gov/pubmed?term=Wyncoll D%5BAuthor%5D&cauthor=true&cauthor_uid=17556722), [Janes J](http://www.ncbi.nlm.nih.gov/pubmed?term=Janes J%5BAuthor%5D&cauthor=true&cauthor_uid=17556722), [Booth FV](http://www.ncbi.nlm.nih.gov/pubmed?term=Booth FV%5BAuthor%5D&cauthor=true&cauthor_uid=17556722), [Wang D](http://www.ncbi.nlm.nih.gov/pubmed?term=Wang D%5BAuthor%5D&cauthor=true&cauthor_uid=17556722), et al. Prophylactic heparin in patients with severe sepsis treated with drotrecogin alfa (activated). [Am J Respir Crit Care Med](http://www.ncbi.nlm.nih.gov/pubmed/17556722#%23) 2007;176(5):483–490.
47. [Eid A](http://www.ncbi.nlm.nih.gov/pubmed?term=Eid A%5BAuthor%5D&cauthor=true&cauthor_uid=18931224), [Wiedermann CJ](http://www.ncbi.nlm.nih.gov/pubmed?term=Wiedermann CJ%5BAuthor%5D&cauthor=true&cauthor_uid=18931224), [Kinasewitz GT](http://www.ncbi.nlm.nih.gov/pubmed?term=Kinasewitz GT%5BAuthor%5D&cauthor=true&cauthor_uid=18931224). Early administration of high-dose antithrombin in severe sepsis: single center results from the KyberSept-trial. [Anesth Analg](http://www.ncbi.nlm.nih.gov/pubmed/?term=2008%09+Early+administration+of+high-dose+antithrombin+in+severe+sepsis+single+center+results+from+the+KyberSept-trial%09Alain+Eid%2C+MD#%23) 2008;107(5):1633-1638.
48. [Zarychanski R](http://www.ncbi.nlm.nih.gov/pubmed?term=Zarychanski R%5BAuthor%5D&cauthor=true&cauthor_uid=18824906), [Doucette S](http://www.ncbi.nlm.nih.gov/pubmed?term=Doucette S%5BAuthor%5D&cauthor=true&cauthor_uid=18824906), [Fergusson D](http://www.ncbi.nlm.nih.gov/pubmed?term=Fergusson D%5BAuthor%5D&cauthor=true&cauthor_uid=18824906), [Roberts D](http://www.ncbi.nlm.nih.gov/pubmed?term=Roberts D%5BAuthor%5D&cauthor=true&cauthor_uid=18824906), [Houston DS](http://www.ncbi.nlm.nih.gov/pubmed?term=Houston DS%5BAuthor%5D&cauthor=true&cauthor_uid=18824906), [Sharma S](http://www.ncbi.nlm.nih.gov/pubmed?term=Sharma S%5BAuthor%5D&cauthor=true&cauthor_uid=18824906), [Gulati H](http://www.ncbi.nlm.nih.gov/pubmed?term=Gulati H%5BAuthor%5D&cauthor=true&cauthor_uid=18824906), [Kumar A](http://www.ncbi.nlm.nih.gov/pubmed?term=Kumar A%5BAuthor%5D&cauthor=true&cauthor_uid=18824906). Early intravenous unfractionated heparin and mortality in septic shock. [Crit Care Med](http://www.ncbi.nlm.nih.gov/pubmed/?term=2008%09Early+intravenous+unfractionated+heparin+and+mortality+in+septic+shock%09Ryan+Zarychanski%2C+MD#%23) 2008;36(11):2973-2979.
49. [Agarwal R](http://www.ncbi.nlm.nih.gov/pubmed?term=Agarwal R%5BAuthor%5D&cauthor=true&cauthor_uid=18250355), [Nath A](http://www.ncbi.nlm.nih.gov/pubmed?term=Nath A%5BAuthor%5D&cauthor=true&cauthor_uid=18250355). XPRESS study: heparin in ... not out. [Am J Respir Crit Care Med](http://www.ncbi.nlm.nih.gov/pubmed/?term=2008%09XPRESS+Study+Heparin+In+Not+Out#%23) 2008;177(4):461; author reply 461-462
50. [Levy M](http://www.ncbi.nlm.nih.gov/pubmed?term=Levy M%5BAuthor%5D&cauthor=true&cauthor_uid=19367397), [Levi M](http://www.ncbi.nlm.nih.gov/pubmed?term=Levi M%5BAuthor%5D&cauthor=true&cauthor_uid=19367397), [Williams MD](http://www.ncbi.nlm.nih.gov/pubmed?term=Williams MD%5BAuthor%5D&cauthor=true&cauthor_uid=19367397), [Antonelli M](http://www.ncbi.nlm.nih.gov/pubmed?term=Antonelli M%5BAuthor%5D&cauthor=true&cauthor_uid=19367397), [Wang D](http://www.ncbi.nlm.nih.gov/pubmed?term=Wang D%5BAuthor%5D&cauthor=true&cauthor_uid=19367397), [Mignini MA](http://www.ncbi.nlm.nih.gov/pubmed?term=Mignini MA%5BAuthor%5D&cauthor=true&cauthor_uid=19367397). Comprehensive safety analysis of concomitant drotrecogin alfa (activated) and prophylactic heparin use in patients with severe sepsis. [Intensive Care Med](http://www.ncbi.nlm.nih.gov/pubmed/?term=2009%09Comprehensive+safety+analysis+of+concomitant+drotrecogin+alfa+(activated)+and+prophylactic+heparin+use+in+patients+with+severe+sepsis%09Mitchell+Levy#%23) 2009;35(7):1196-1203.
51. [Angstwurm M](http://www.ncbi.nlm.nih.gov/pubmed?term=Angstwurm M%5BAuthor%5D&cauthor=true&cauthor_uid=19189066), [Hoffmann J](http://www.ncbi.nlm.nih.gov/pubmed?term=Hoffmann J%5BAuthor%5D&cauthor=true&cauthor_uid=19189066), [Ostermann H](http://www.ncbi.nlm.nih.gov/pubmed?term=Ostermann H%5BAuthor%5D&cauthor=true&cauthor_uid=19189066), [Frey L](http://www.ncbi.nlm.nih.gov/pubmed?term=Frey L%5BAuthor%5D&cauthor=true&cauthor_uid=19189066), [Spannagl M](http://www.ncbi.nlm.nih.gov/pubmed?term=Spannagl M%5BAuthor%5D&cauthor=true&cauthor_uid=19189066). Severe sepsis and disseminated intravascular coagulation. Supplementation with antithrombin [Anaesthesist](http://www.ncbi.nlm.nih.gov/pubmed/?term=2009%09Severe+sepsis+and+disseminated+intravascular+coagulation.+Supplementation+with+antithrombin%09M.+Angstwurm#%23) 2009;58(2):171-179.
52. [Tsen A](http://www.ncbi.nlm.nih.gov/pubmed?term=Tsen A%5BAuthor%5D&cauthor=true&cauthor_uid=18636042), [Kirschenbaum LA](http://www.ncbi.nlm.nih.gov/pubmed?term=Kirschenbaum LA%5BAuthor%5D&cauthor=true&cauthor_uid=18636042), [LaRow C](http://www.ncbi.nlm.nih.gov/pubmed?term=LaRow C%5BAuthor%5D&cauthor=true&cauthor_uid=18636042), [Khan R](http://www.ncbi.nlm.nih.gov/pubmed?term=Khan R%5BAuthor%5D&cauthor=true&cauthor_uid=18636042), [Kurtz S](http://www.ncbi.nlm.nih.gov/pubmed?term=Kurtz S%5BAuthor%5D&cauthor=true&cauthor_uid=18636042), [Bansal S](http://www.ncbi.nlm.nih.gov/pubmed?term=Bansal S%5BAuthor%5D&cauthor=true&cauthor_uid=18636042), [Astiz ME](http://www.ncbi.nlm.nih.gov/pubmed?term=Astiz ME%5BAuthor%5D&cauthor=true&cauthor_uid=18636042). The effect of anticoagulants and the role of thrombin on neutrophil-endothelial cell interactions in septic shock. [Shock](http://www.ncbi.nlm.nih.gov/pubmed/?term=2009%09The+effect+of+anticoagulants+and+the+role+of+thrombin+on+neutrophil-endothelial+cell+interactions+in+septic+shock%09Anita+Tsen%2C+Linda+A#%23) 2009;31(2):120-124.
53. [Shorr AF](http://www.ncbi.nlm.nih.gov/pubmed?term=Shorr AF%5BAuthor%5D&cauthor=true&cauthor_uid=19132200), [Williams MD](http://www.ncbi.nlm.nih.gov/pubmed?term=Williams MD%5BAuthor%5D&cauthor=true&cauthor_uid=19132200). Venous thromboembolism in critically ill patients. Observations from a randomized trial in sepsis. [Thromb Haemost](http://www.ncbi.nlm.nih.gov/pubmed/?term=2009%09Venous+thromboembolism+in+critically+ill+patients.+Observations+from+a+randomized+trial+in+sepsis%09Andrew+F.Shorr#%23) 2009;101(1):139-144.
54. [Iba T](http://www.ncbi.nlm.nih.gov/pubmed?term=Iba T%5BAuthor%5D&cauthor=true&cauthor_uid=22542365), [Saito D](http://www.ncbi.nlm.nih.gov/pubmed?term=Saito D%5BAuthor%5D&cauthor=true&cauthor_uid=22542365), [Wada H](http://www.ncbi.nlm.nih.gov/pubmed?term=Wada H%5BAuthor%5D&cauthor=true&cauthor_uid=22542365), [Asakura H](http://www.ncbi.nlm.nih.gov/pubmed?term=Asakura H%5BAuthor%5D&cauthor=true&cauthor_uid=22542365). Efficacy and bleeding risk of antithrombin supplementation in septic disseminated intravascular coagulation: a prospective multicenter survey. [Thromb Res](http://www.ncbi.nlm.nih.gov/pubmed/?term=Efficacy+and+bleeding+risk+of+antithrombin+supplementation+in+septic+disseminatedintravascular+coagulation%3A+A+prospective+multicenter+survey#%23) 2012;130(3):e129–133.
55. [Han XD](http://www.ncbi.nlm.nih.gov/pubmed?term=Han XD%5BAuthor%5D&cauthor=true&cauthor_uid=22248749), [Sun H](http://www.ncbi.nlm.nih.gov/pubmed?term=Sun H%5BAuthor%5D&cauthor=true&cauthor_uid=22248749), [Huang XY](http://www.ncbi.nlm.nih.gov/pubmed?term=Huang XY%5BAuthor%5D&cauthor=true&cauthor_uid=22248749), [Zhang SY](http://www.ncbi.nlm.nih.gov/pubmed?term=Zhang SY%5BAuthor%5D&cauthor=true&cauthor_uid=22248749), [Wang YD](http://www.ncbi.nlm.nih.gov/pubmed?term=Wang YD%5BAuthor%5D&cauthor=true&cauthor_uid=22248749), [Ren K](http://www.ncbi.nlm.nih.gov/pubmed?term=Ren K%5BAuthor%5D&cauthor=true&cauthor_uid=22248749), [Li F](http://www.ncbi.nlm.nih.gov/pubmed?term=Li F%5BAuthor%5D&cauthor=true&cauthor_uid=22248749). A clinical study of pituitrin versus norepinephrine in the treatment of patients with septic shock [Zhongguo Wei Zhong Bing Ji Jiu Yi Xue](http://www.ncbi.nlm.nih.gov/pubmed/?term=2012%09A+clinical+study+of+pituitrln+versus+norepinephrine+in+the+treatment+of+patients+with+septic+shock+%09HAN+Xu-dong#%23) 2012;24(1):33-37.
56. [Gamzatov KhA](http://www.ncbi.nlm.nih.gov/pubmed?term=Gamzatov KhA%5BAuthor%5D&cauthor=true&cauthor_uid=23227740), [Gurzhiĭ DV](http://www.ncbi.nlm.nih.gov/pubmed?term=Gurzhiĭ DV%5BAuthor%5D&cauthor=true&cauthor_uid=23227740), [Lazarev SM](http://www.ncbi.nlm.nih.gov/pubmed?term=Lazarev SM%5BAuthor%5D&cauthor=true&cauthor_uid=23227740), [Nemestnikov IuA](http://www.ncbi.nlm.nih.gov/pubmed?term=Nemestnikov IuA%5BAuthor%5D&cauthor=true&cauthor_uid=23227740), [Gurzhiĭ AA](http://www.ncbi.nlm.nih.gov/pubmed?term=Gurzhiĭ AA%5BAuthor%5D&cauthor=true&cauthor_uid=23227740), [Panaian LP](http://www.ncbi.nlm.nih.gov/pubmed?term=Panaian LP%5BAuthor%5D&cauthor=true&cauthor_uid=23227740), [Golovina OG](http://www.ncbi.nlm.nih.gov/pubmed?term=Golovina OG%5BAuthor%5D&cauthor=true&cauthor_uid=23227740), [Khait EA](http://www.ncbi.nlm.nih.gov/pubmed?term=Khait EA%5BAuthor%5D&cauthor=true&cauthor_uid=23227740), [Smirnova OA](http://www.ncbi.nlm.nih.gov/pubmed?term=Smirnova OA%5BAuthor%5D&cauthor=true&cauthor_uid=23227740), [Matvienko OIu](http://www.ncbi.nlm.nih.gov/pubmed?term=Matvienko OIu%5BAuthor%5D&cauthor=true&cauthor_uid=23227740). Assessment of endogenous thrombin potential and influence on it of different regimens of heparin therapy in patients with abdominal sepsis. [Vestn Khir Im I I Grek.](http://www.ncbi.nlm.nih.gov/pubmed/?term=Assessment+of+endogenous+thrombin+potential+and+influence+on+it+of+differentregimens+of+heparin+therapy+in+patients+with+abdominal+sepsisVestn+Khir+Im+I+I+Grek.+2012%3B171(5)%3A31-6.#%23) 2012;171(5):31-36.

Additional file 2 Meta-analysis of high quality articles

After excluding low quality studies[7, 8, 28]，Six studies (Table 1) were included in the meta-analysis [4, 23-28]. Except for a lack of 28-day mortality data in Yang’s study [27], the remaining five studies all performed analyses of 28-day mortality. Raw data were directly provided in five of the six studies [4,23-25,27]. The data from one study [26] were obtained indirectly from the corresponding authors [29]. The abstract of Abraham’s study [25] indicated that 28-day mortality was the primary outcome; however, the specific time point was not shown in the results and tables. We included this study for 28-day mortality analysis after discussion. Five studies [4,23-25,27] were included in the meta-analysis of bleeding events.

**28-day mortality analysis**

We performed analyses for five studies [4,23-26], which included 3383 participants (2323 participants were included in the patient group and 1060 participants in the control group). Within 28 days of admission, 698 (30.04%) died in the patient group, and 397 (37.45%) died in the control group (OR = 0.653; 95% CI = 0.558-0.764; P < 0.0001; I^2^ = 0.0%), indicating a statistically significant reduction in 28-day mortality in heparin-treated patients with sepsis (see Figure 2-1). There was no evidence of between-study heterogeneity (I^2^=0.0%), and a sensitivity analysis was not performed.


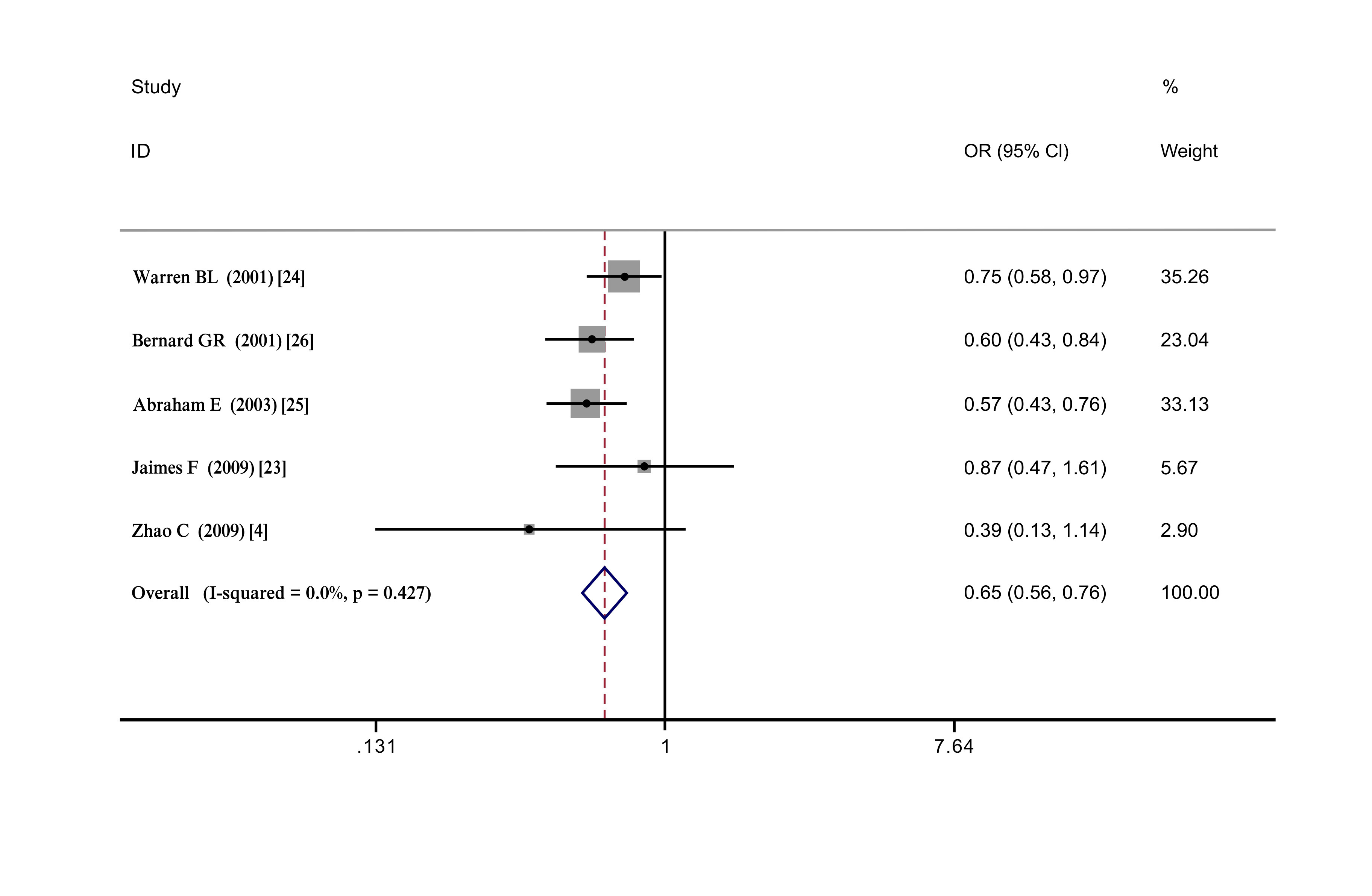


Figure 2-1 Forest plot of 28-day mortality

**Subgroup analysis**. Subgroup analysis was performed according to the different experimental designs of the five studies. The included studies were divided into two subgroups: an NRCT group and an RCT group. For the three NRCT studies, OR = 0.648, 95% CI = 0.550 -0.764, P < 0.001, and I^2^ = 6.7%. For the two RCT studies, OR = 0.707, 95% CI = 0.413 -1.201, P = 0.198, and I^2^ = 0.0%. Subgroup analysis indicated that the results of the NRCT group reached statistical significance. Although the results from the RCT group are not statistically significant, the high value of the 95% CIs (1.201) is very close to the invalid line. When heterogeneity was assessed in the different subgroups, there was either low-level or no heterogeneity (see Figure 2-2).


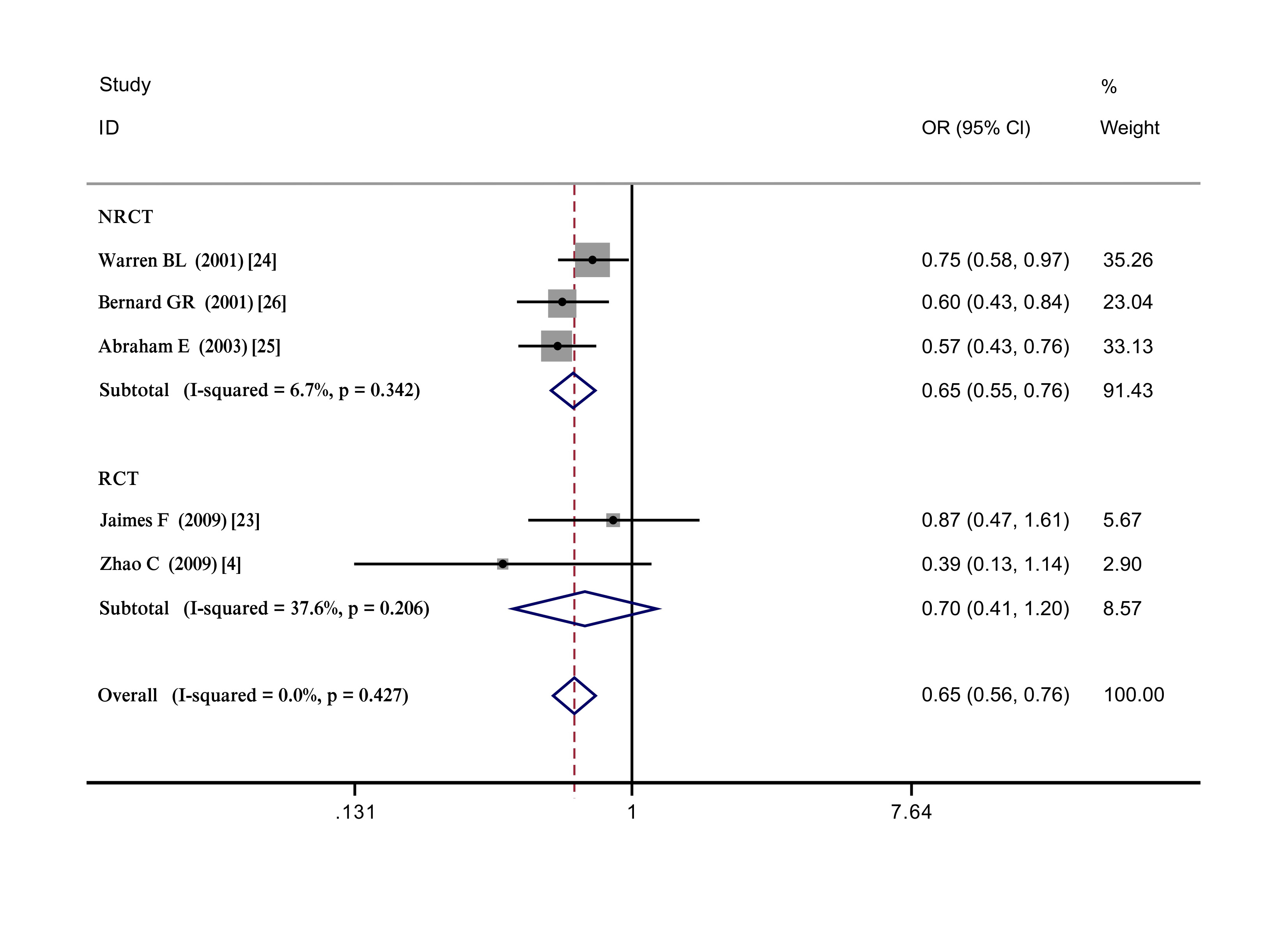


Figure 2-2 Subgroup analysis of 28-day mortality(according to the different experimental designs)

Subgroup analysis was also performed according to sepsis severity. For the there studies on severe sepsis (defined as sepsis complicated by organ dysfunction and tissue hypoperfusion), OR = 0.648, 95% CI = 0.550 -0.764, P < 0.001, and I^2^ = 6.7%. For the two studies on non-severe sepsis, OR = 0.707, 95% CI = 0.413 -1.201, P = 0.198, and I^2^ = 0.0%. Heparin may have therapeutic effects in patients with severe sepsis. Similar to the subgroup analysis of the different experimental designs, the high 95% CIs from the sepsis group were also very close to the invalid line (see Figure 2-3).


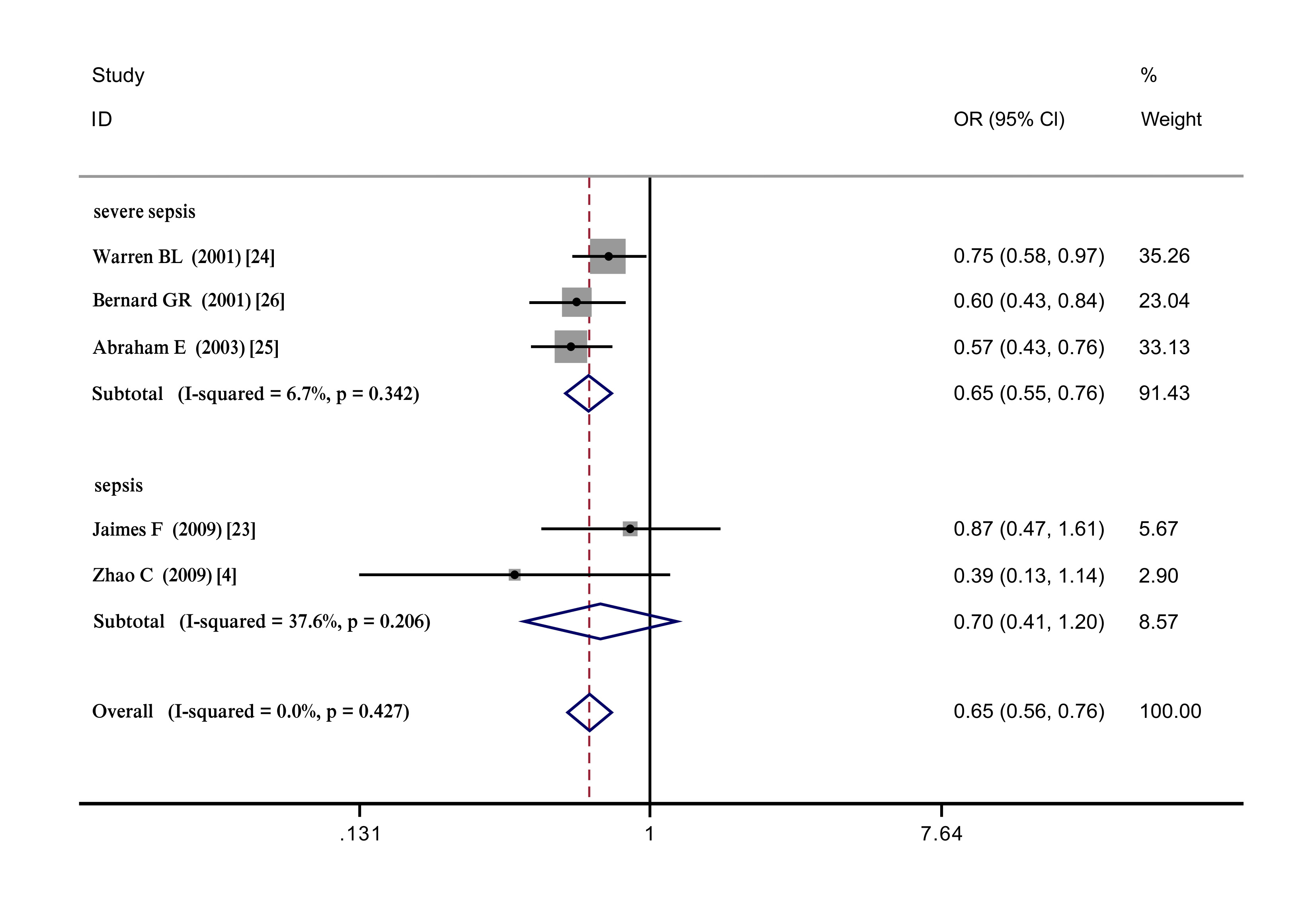


Figure 2-3 Subgroup analysis of 28-day mortality(according to sepsis severity)

**Publication bias analysis**. We also analyzed publication bias for 28-day mortality in the included studies. Because the response variables were dichotomous, publication bias was quantitatively examined using the Harbord test. The P-value was 0.753 in the 28-day mortality analysis, indicating that there was no evidence of publication bias in these studies (see Figure 2-4).


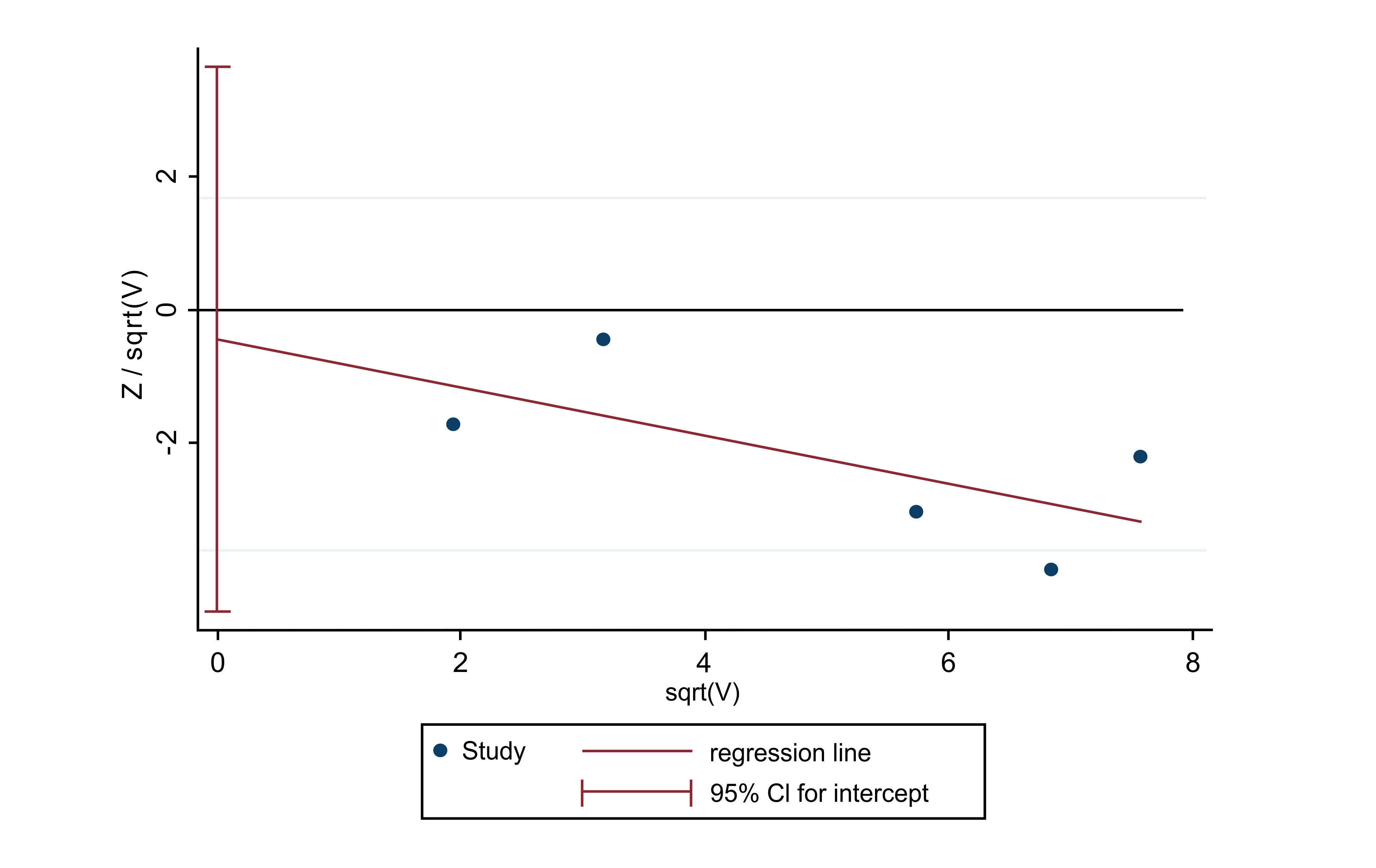


Figure 2-4 The Harbord plot for 28-day mortality

**The bleeding events analysis**

We performed a statistical analysis on five studies [4, 23-26] that included 2664 participants (1742 participants in the patient group and 922 participants in the control group). There were 247 (14.17%) bleeding events in the patient group and 112 (12.14%) in the control group (OR = 1.035; 95% CI = 0.811-1.322; P = 0.782; I^2^ = 7.0%). The results failed to reach statistical significance, indicating that heparin has no effect on bleeding events in patients with sepsis (see Figure 2-5).


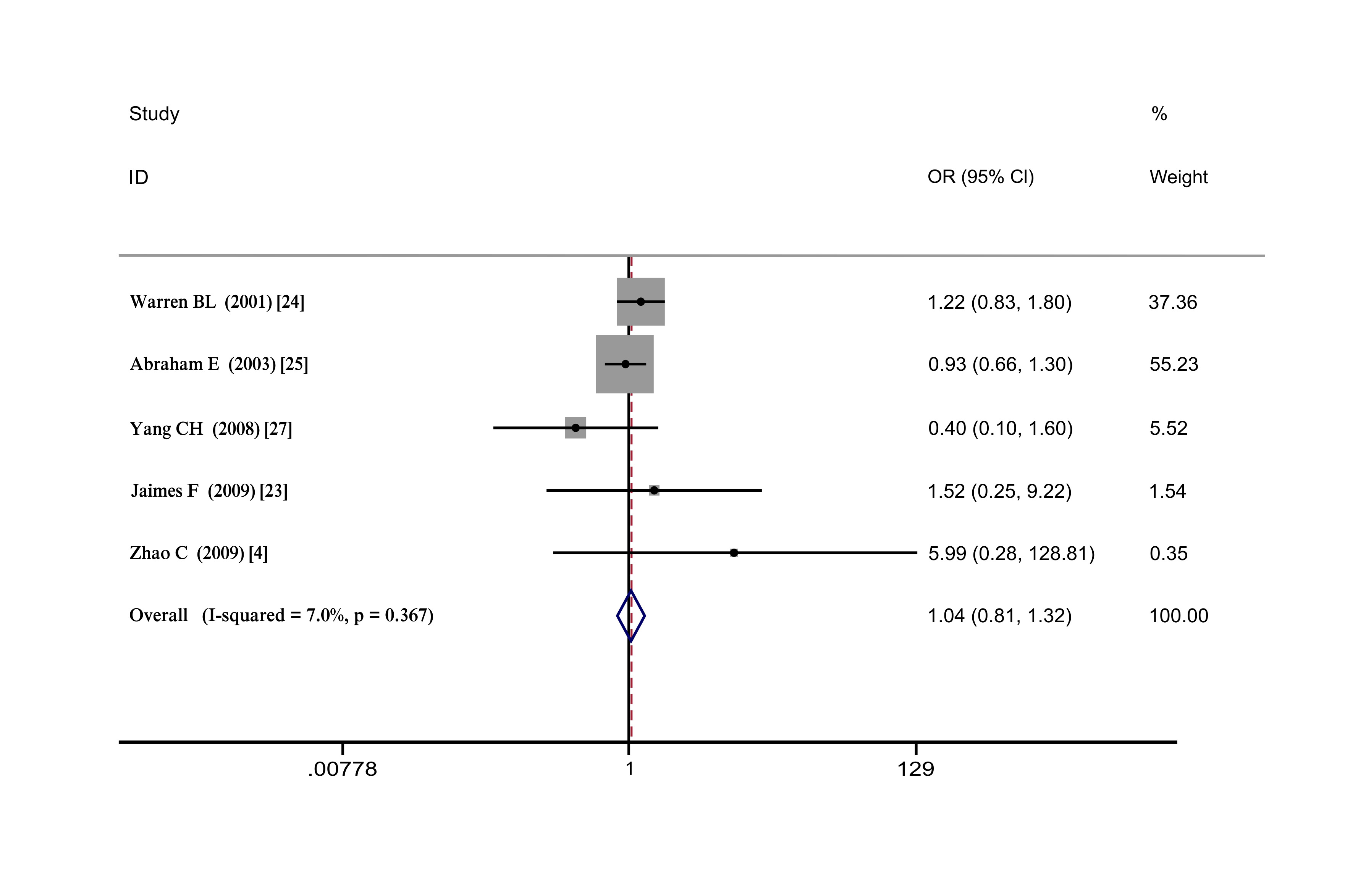


Figure 2-5 Forest plot of bleeding events

There was a low level of heterogeneity between studies, and a sensitivity analysis was not performed.

**Subgroup analysis.** To investigate whether the bleeding events were associated with sepsis severity, we conducted a subgroup analysis according to sepsis severity. For the severe sepsis group, OR = 1.046, 95% CI= 0.812 -1.349, P = 0.569, I^2^ = 7.4%; for the sepsis group, OR = 0.897, 95%CI = 0.354 -2.275, P = 0.819, I^2^ = 35.1%. The statistical results did not show that bleeding events were related to sepsis severity (see Figure 2-6). Subgroup analysis was performed to investigate the effects of different subgroups on heterogeneity. The included studies were divided into two subgroups: an NRCT group and an RCT group. For the two NRCT studies, OR= 1.046, 95%CI = 0.86 -1.36, P = 0.727, I^2^ = 7.4%. For the three RCT studies, OR = 0.89, 95%CI = 0.354 - 2.275, P = 0.819, and I^2^ = 35.1%. Four studies with sample sizes over 100 were included in this subgroup with an OR = 1.018, 95%CI = 0.796 - 1.302，P = 0.887, and I^2^ = 1.0%. Only one studies was included in the subgroup with sample sizes less than 100 with an OR =5.986，95%CI =0.0.278 -128.805，P =0.782. (see Figures 2-7, 2-8).


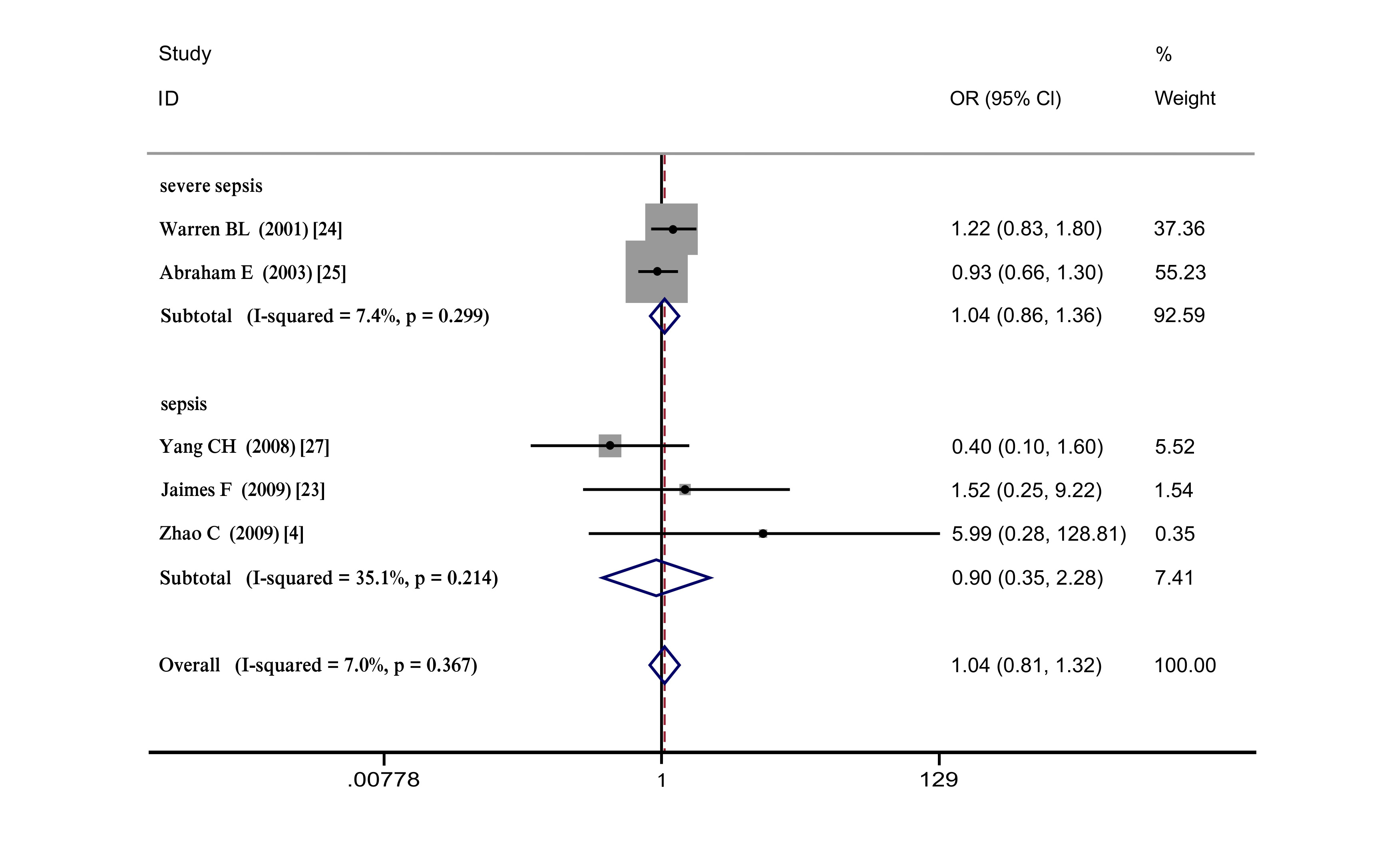


Figure 2-6 Subgroup analysis hemorrhagic events(according to sepsis severity)


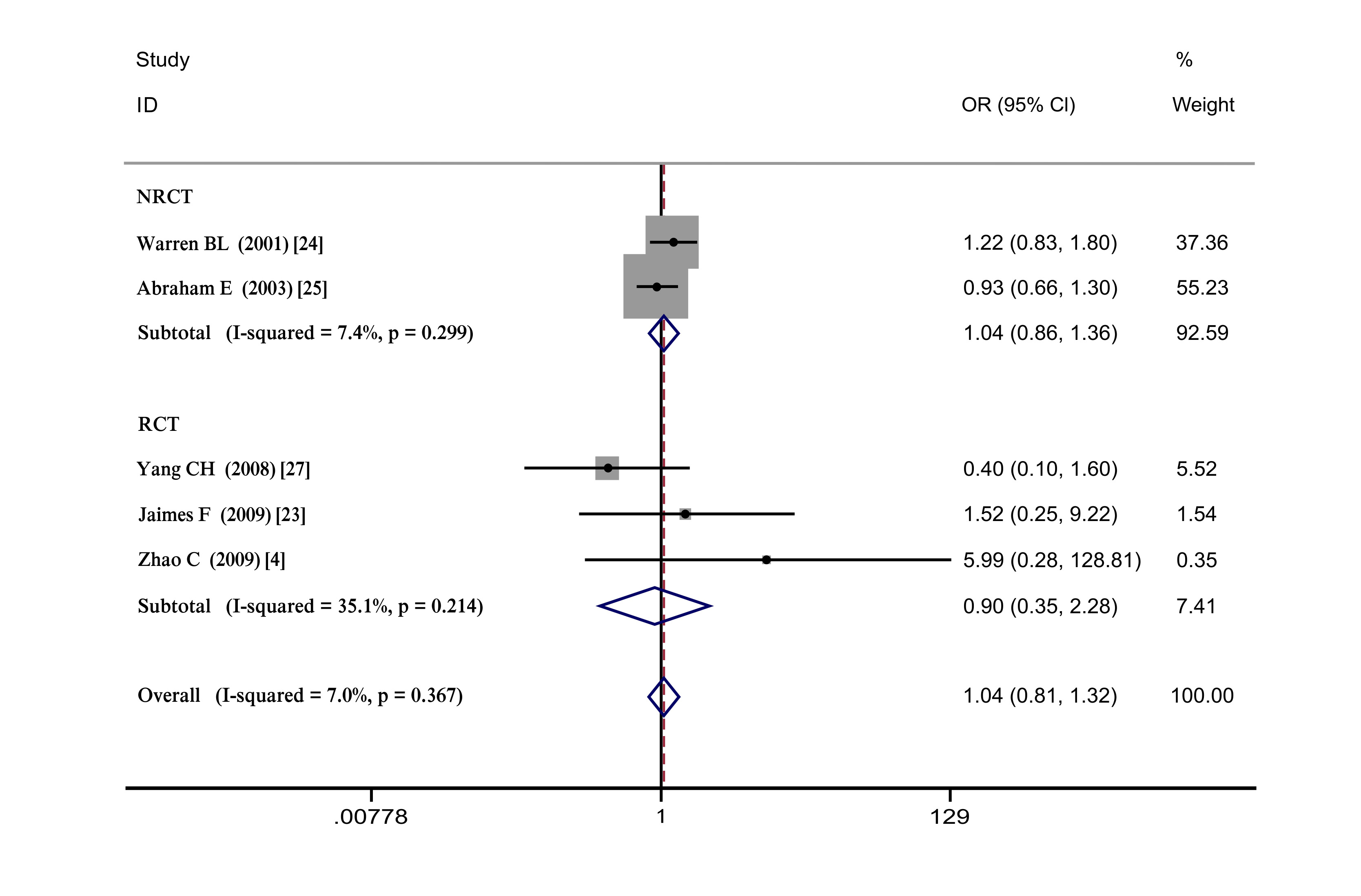


Figure 2-7 Subgroup analysis hemorrhagic events(according to the different

experimental designs)


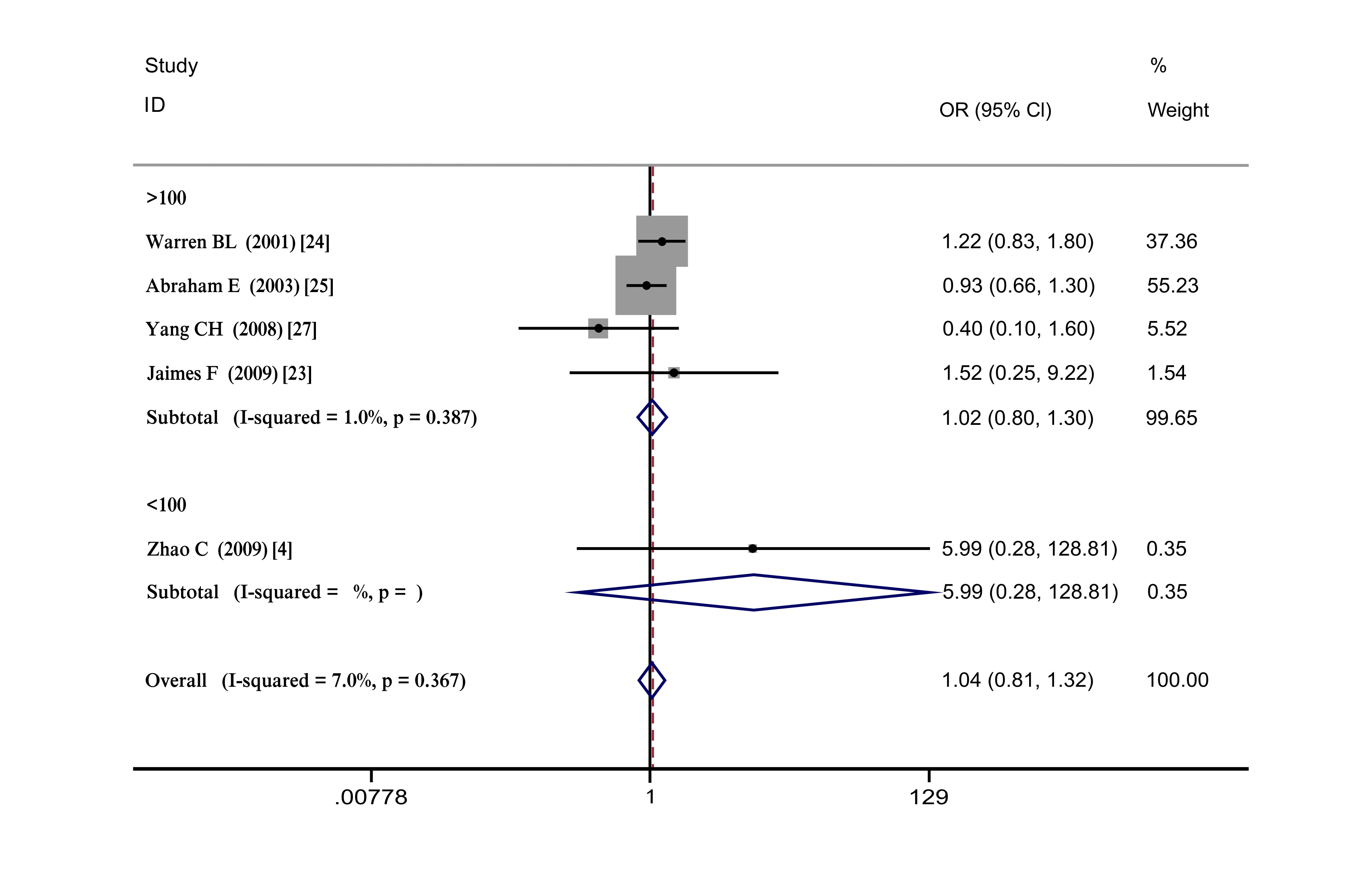


Figure 2-8 Subgroup analysis hemorrhagic events(according to sample size)

**Publication bias analysis**. The P-value was 0.701 in the bleeding events analysis (see Figure 2-9).


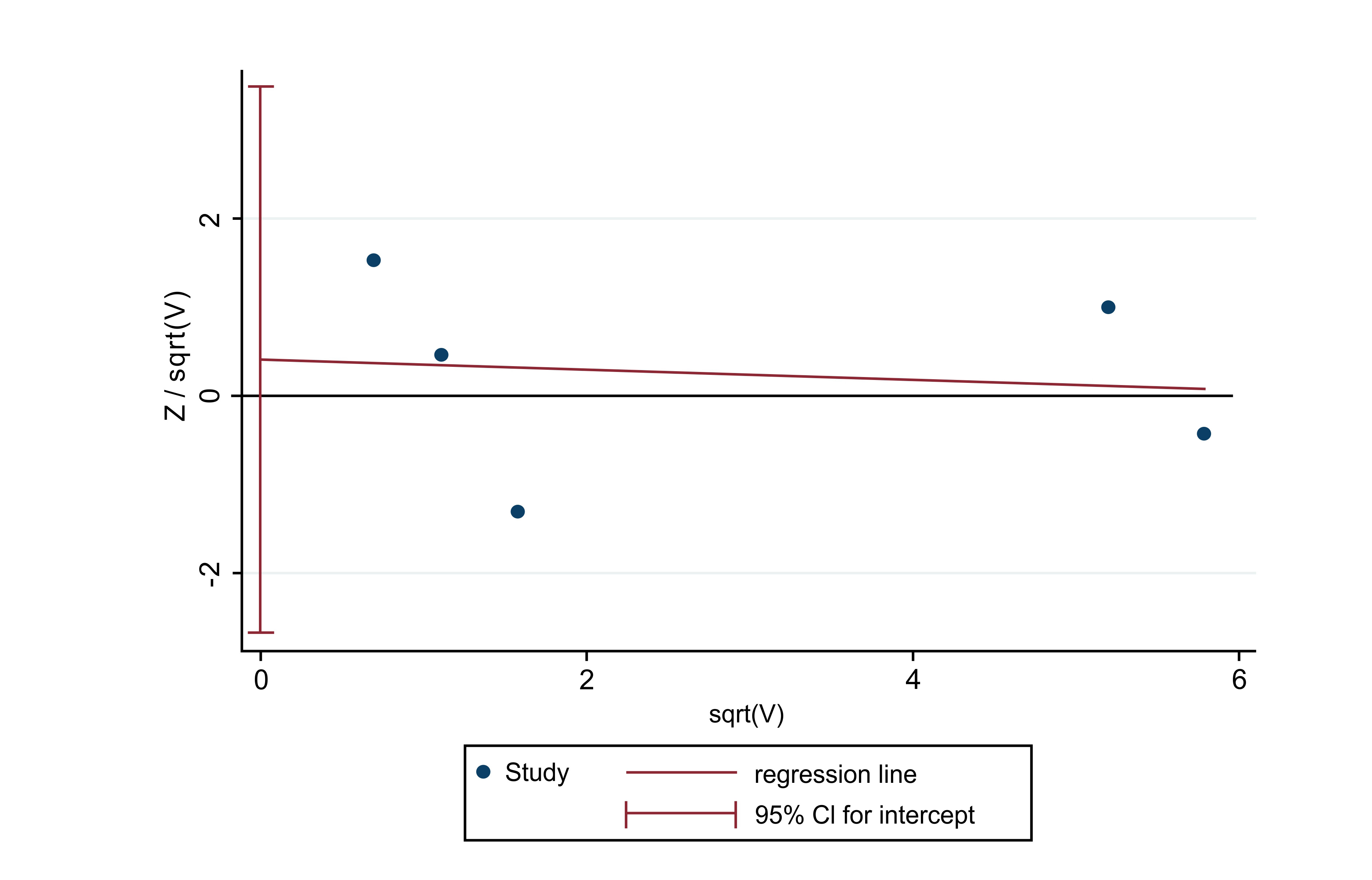


Figure 2-9 The Harbord plot for hemorrhagic events

The results of high quality articles have no difference with the results of all articles.

Additional file 2-1 Forest plot of 28-day mortality

Additional file 2-2 Subgroup analysis of 28-day mortality(according to the different experimental designs)

Additional file 2-3 Subgroup analysis of 28-day mortality(according to sepsis severity)

Additional file 2-4 The Harbord plot for 28-day mortality

Additional file 2-5 Forest plot of bleeding events

Additional file 2-6 Subgroup analysis hemorrhagic events(according to sepsis severity)

Additional file 2-7 Subgroup analysis hemorrhagic events(according to the different

experimental designs)

Additional file 2-8 Subgroup analysis hemorrhagic events(according to sample size)

Additional file 2-9 The Harbord plot for hemorrhagic events

Additional file 3 Outcome effect estimates

| Outcome | N | OR(95%Cl) | *P*_OR_ | *I^2^* (*P*) | *P*_harbord_ |
| --- | --- | --- | --- | --- | --- |
| 28-Day Morality |  |  |  |  |  |
| Total | 8 | 0.656(0.562,0.765) | <0.001 | 0.0% (0.772) | 0.881 |
| NRCT | 3 | 0.648(0.550,0.764) | <0.001 | 6.7% (0.342) | 0.543 |
| RCT | 5 | 0.717(0.458,1.122) | 0.145 | 0.0% (0.786) | 0.722 |
| Severe sepsis | 4 | 0.650(0.552,0.766) | <0.001 | 0.0%(0.503) | 0.839 |
| Sepsis | 4 | 0.702(0.443,1.115) | 0.134 | 0.0%(0.659) | 0.470 |
| Bleeding event |  |  |  |  |  |
| total | 7 | 1.063(0.834,1.355) | 0.623 | 20.9%(0.277) | 0.260 |
| Sample size<100 | 3 | 7.497(0.885,63.529) | 0.065 | 0.0% (0.852) | - |
| Sample size>100 | 4 | 1.018(0.796,1.302) | 0.887 | 1.0% (0.387) | 0.722 |
| NRCT | 2 | 1.045(0.802,1.362) | 0.745 | 7.4% (0.299) | - |
| RCT | 5 | 1.524(0.369,6.292) | 0.561 | 43.4%(0.151) | 0.308 |
|  |  |  |  |  |  |
| Severe sepsis | 3 | 1.076(0.836 ,1.384) | 0.569 | 34.9%(0.215) | - |
| Sepsis | 4 | 0.90(0.35,2.28) | 0.82 | 35.1%(0.214) | 0.062 |

Additional file 4. Forest plot of 28-day mortality

Additional file 5. Subgroup analysis of 28-day mortality(according to the different experimental designs)

Additional file 6. The Harbord plot for 28-day mortality

Additional file 7. Subgroup analysis hemorrhagic events(according to sepsis severity)Additional file 8. Subgroup analysis hemorrhagic events(according to the different

experimental designs)

Additional file 9. Subgroup analysis hemorrhagic events(according to sample size)

Additional file 10. The Harbord plot for hemorrhagic events
