# Supplementary figures and images for: Heparin therapy reduces 28-day mortality in adult severe sepsis patients: a systematic review and meta-analysis
Source: Crit Care. 2014 Oct 16;18(5):563. doi: 10.1186/s13054-014-0563-4 (PMC4213495; doi:10.1186/s13054-014-0563-4)

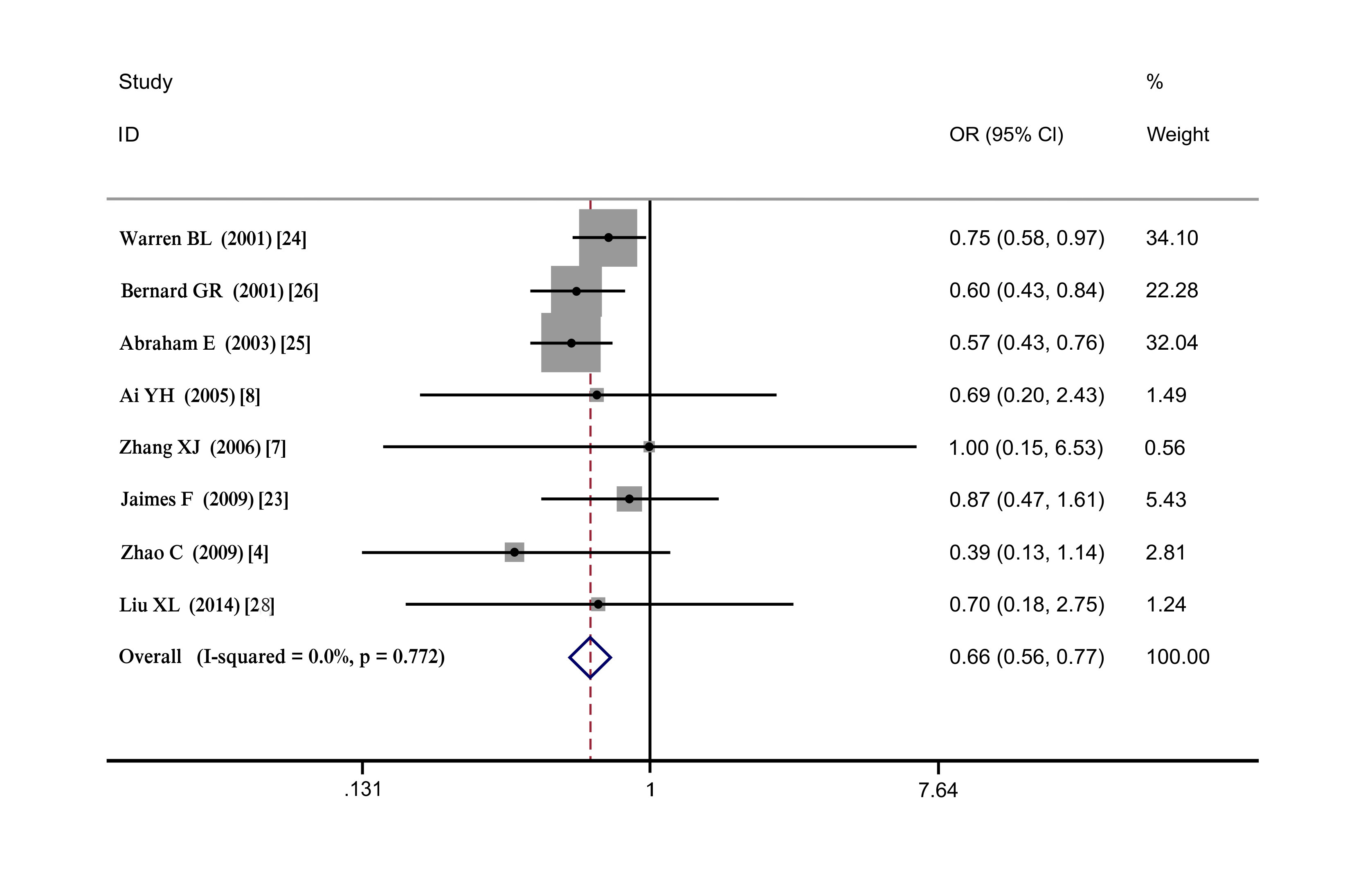

Supplement: Additional file 2: — Forest plot of 28-day mortality. [file 13054_2014_563_MOESM2_ESM.jpeg]

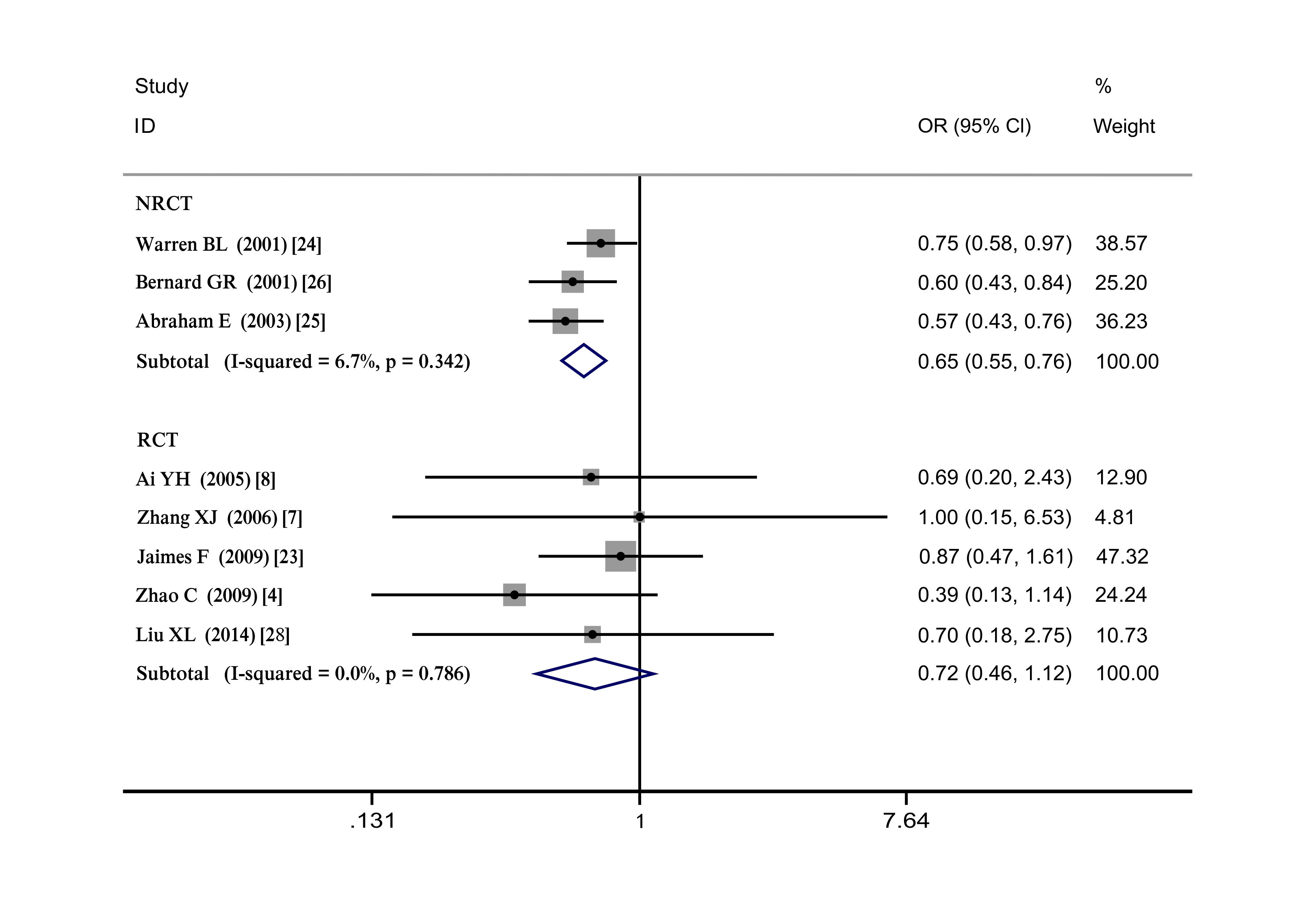

Supplement: Additional file 3: — Subgroup analysis of 28-day mortality (according to the different experimental designs). [file 13054_2014_563_MOESM3_ESM.jpeg]

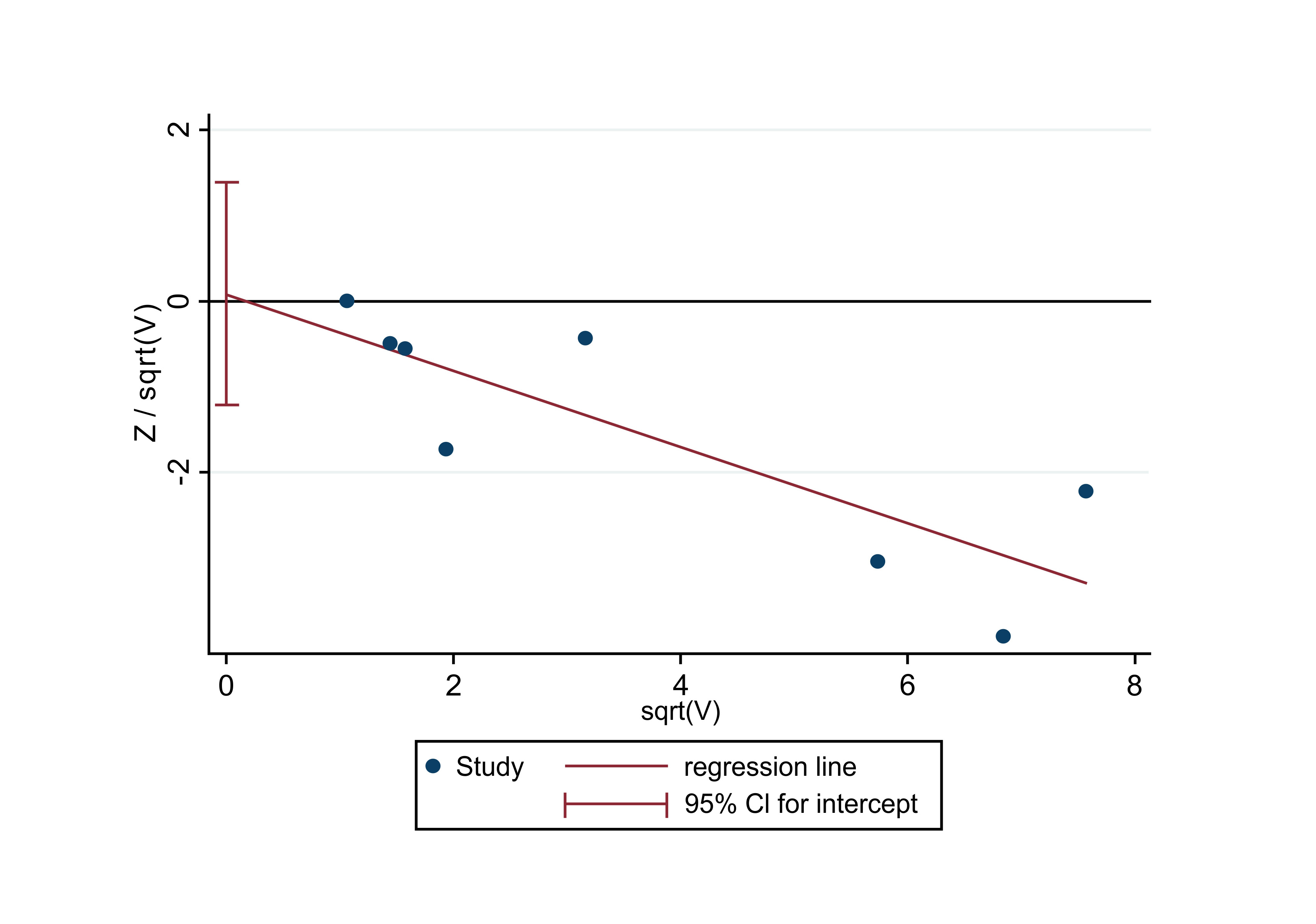

Supplement: Additional file 4: — The Harbord plot for 28-day mortality. [file 13054_2014_563_MOESM4_ESM.jpeg]

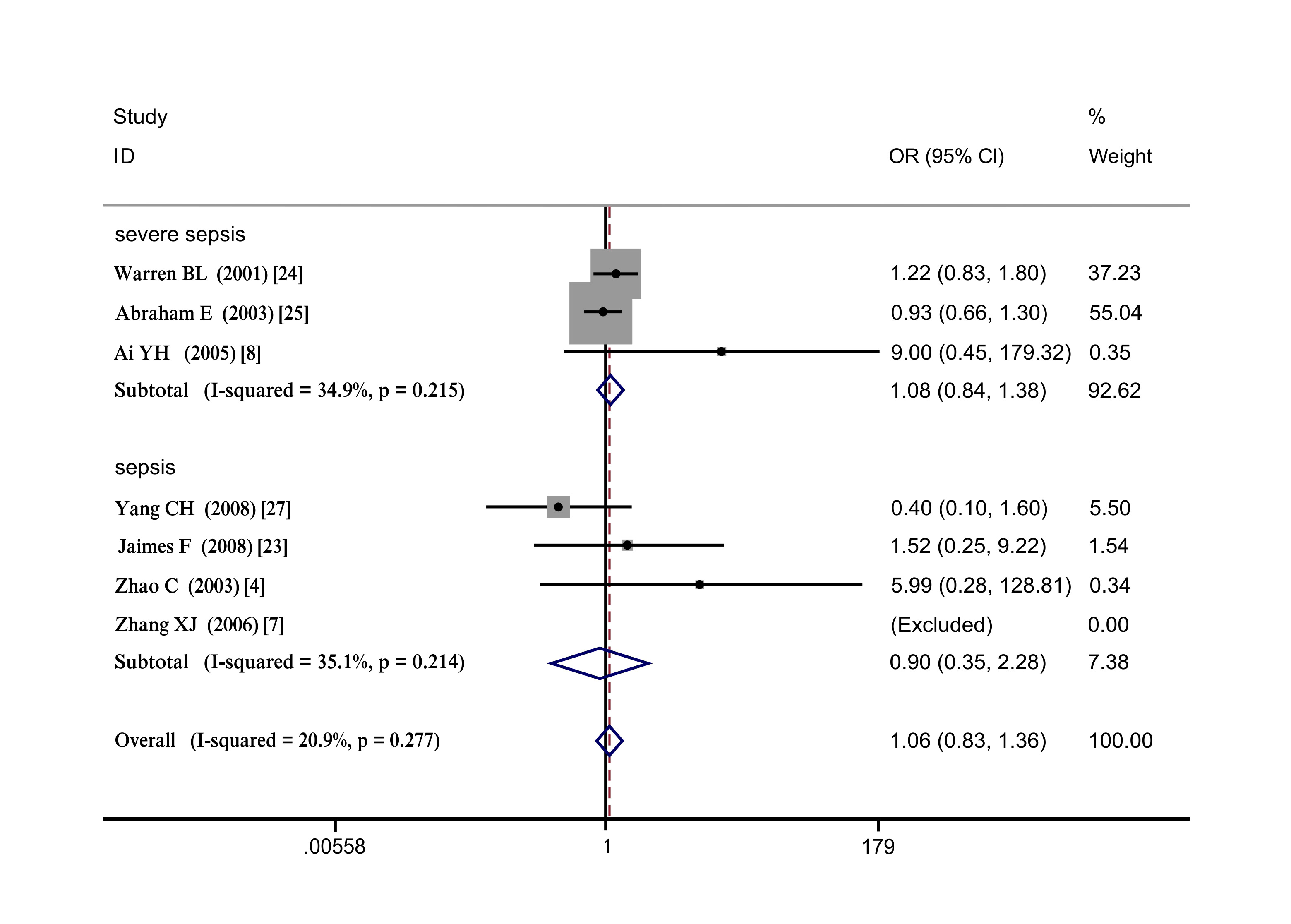

Supplement: Additional file 5: — Subgroup analysis hemorrhagic events (according to sepsis severity). [file 13054_2014_563_MOESM5_ESM.jpeg]

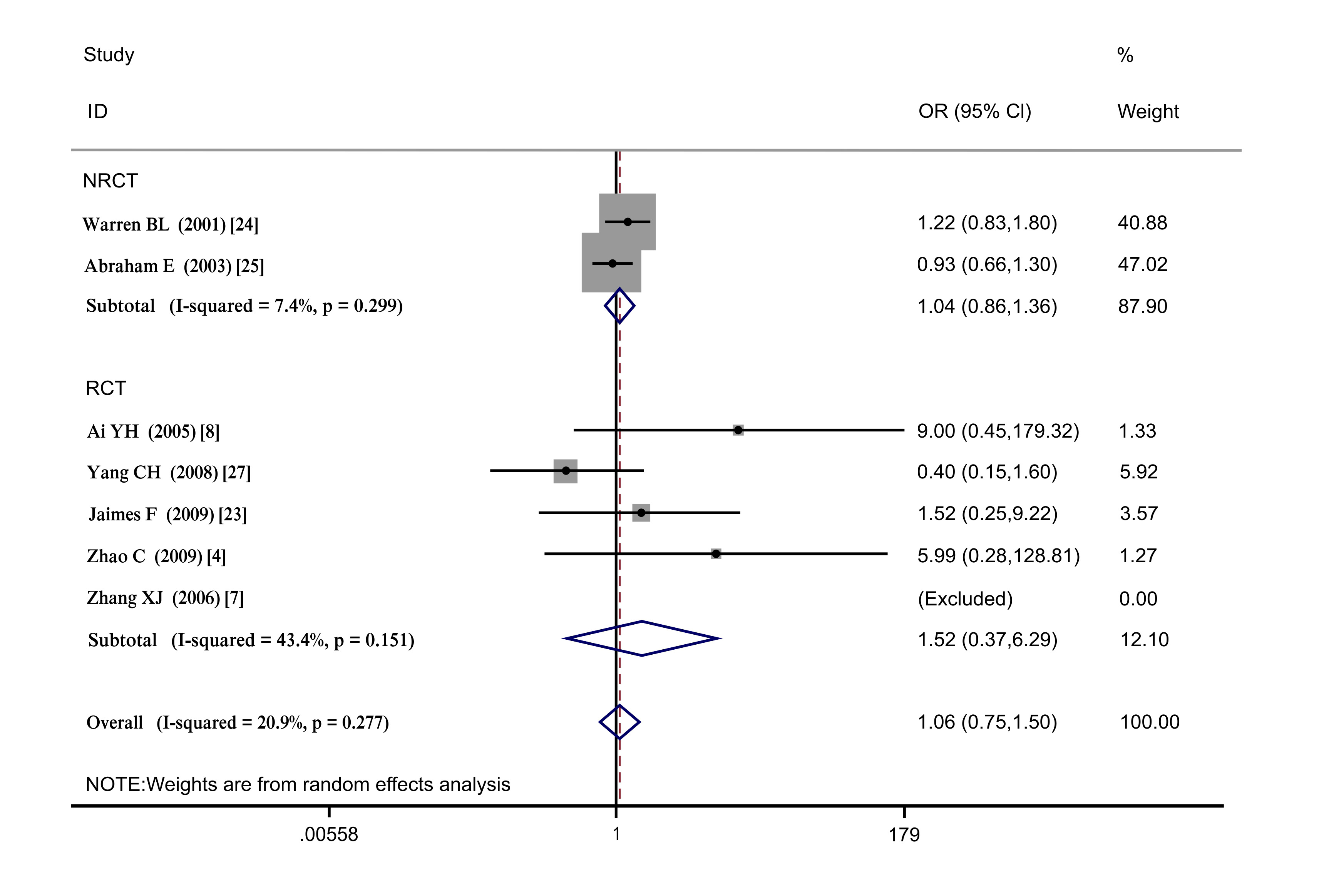

Supplement: Additional file 6: — Subgroup analysis hemorrhagic events (according to the different experimental designs). [file 13054_2014_563_MOESM6_ESM.jpeg]

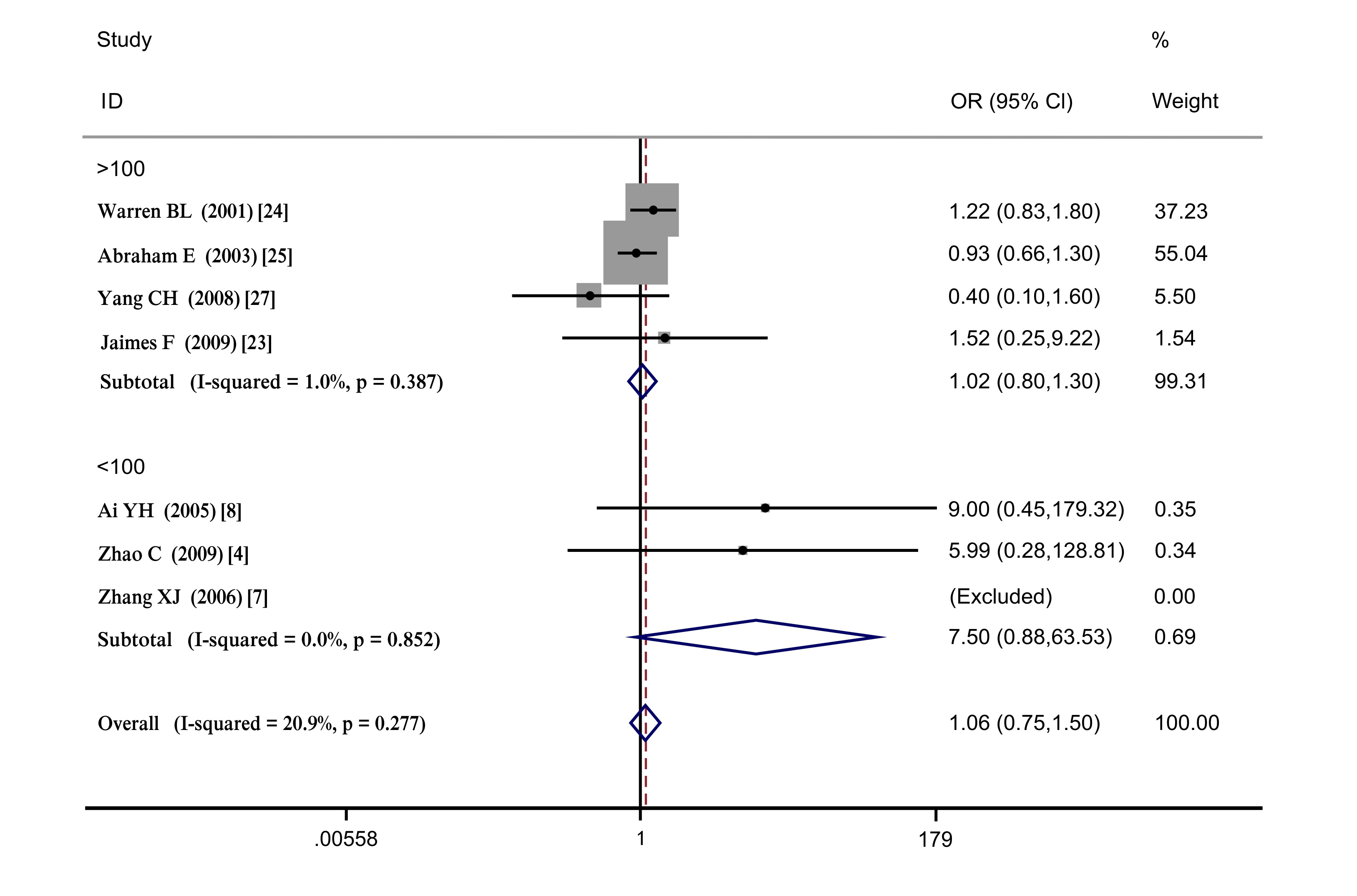

Supplement: Additional file 7: — Subgroup analysis hemorrhagic events (according to sample size). [file 13054_2014_563_MOESM7_ESM.jpeg]

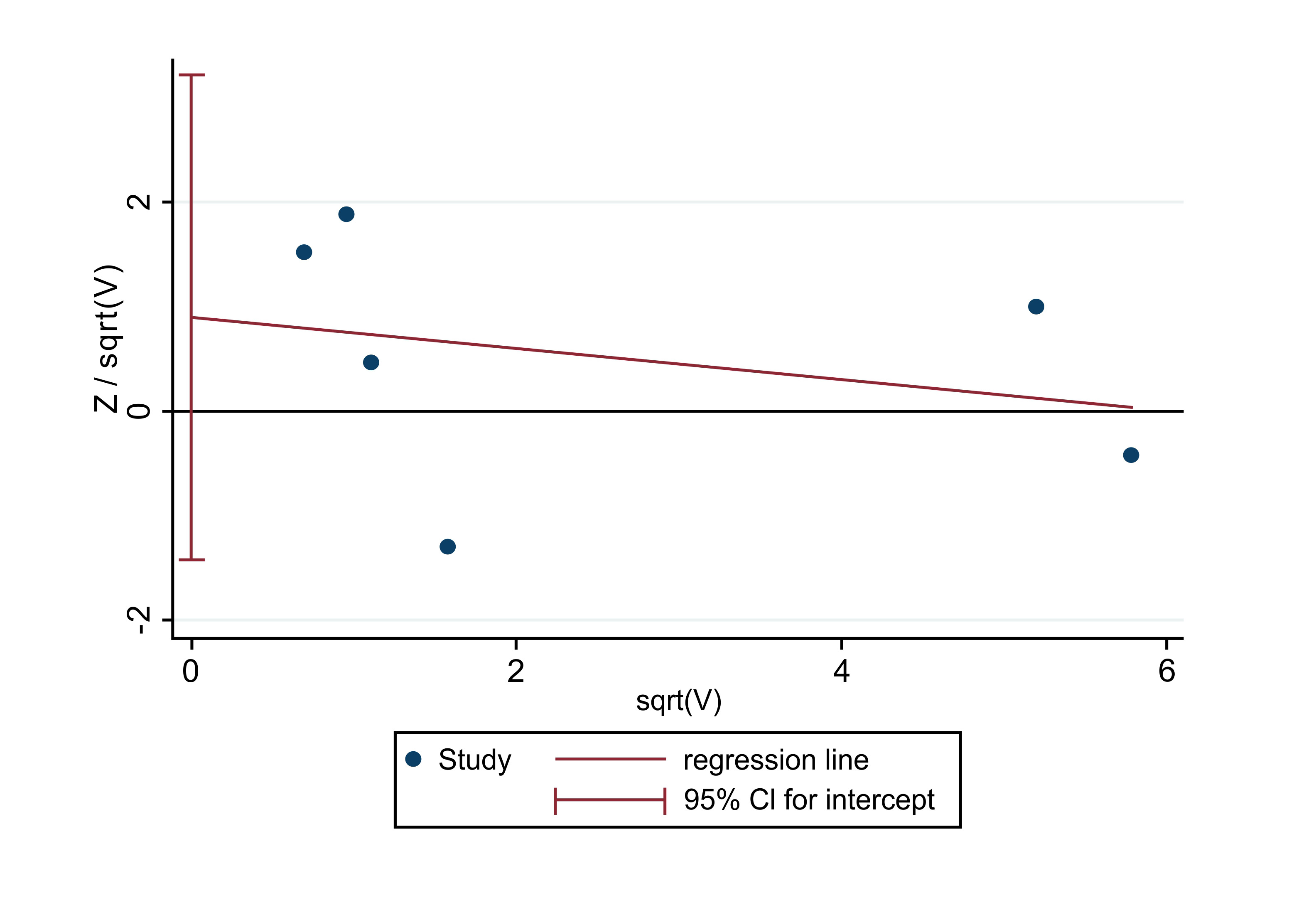

Supplement: Additional file 8: — The Harbord plot for hemorrhagic events. [file 13054_2014_563_MOESM8_ESM.jpeg]
